# Pattern statistics on Markov chains and sensitivity to parameter estimation

**DOI:** 10.1186/1748-7188-1-17

**Published:** 2006-10-17

**Authors:** Grégory Nuel

**Affiliations:** 1 Laboratoire Statistique et Génome, University of Evry, CNRS (8071), INRA(1152), 523, place des terrasses de I'Agora, 91034 Evry CEDEX, France

## Abstract

**Background:**

In order to compute pattern statistics in computational biology a Markov model is commonly used to take into account the sequence composition. Usually its parameter must be estimated. The aim of this paper is to determine how sensitive these statistics are to parameter estimation, and what are the consequences of this variability on pattern studies (finding the most over-represented words in a genome, the most significant common words to a set of sequences,...).

**Results:**

In the particular case where pattern statistics (overlap counting only) computed through binomial approximations we use the delta-method to give an explicit expression of *σ*, the standard deviation of a pattern statistic. This result is validated using simulations and a simple pattern study is also considered.

**Conclusion:**

We establish that the use of high order Markov model could easily lead to major mistakes due to the high sensitivity of pattern statistics to parameter estimation.

## Background

In order to study pattern occurrences in biological sequences, simple frequencies are not relevant in most cases because of pattern overlapping structure as well as composition bias in the sequences. A common workaround consists to compute the significance of an observation assuming the sequence *X *= *X*_1 _... *X*_ℓ _over the finite alphabet A
 MathType@MTEF@5@5@+=feaafiart1ev1aaatCvAUfKttLearuWrP9MDH5MBPbIqV92AaeXatLxBI9gBamrtHrhAL1wy0L2yHvtyaeHbnfgDOvwBHrxAJfwnaebbnrfifHhDYfgasaacH8akY=wiFfYdH8Gipec8Eeeu0xXdbba9frFj0=OqFfea0dXdd9vqai=hGuQ8kuc9pgc9s8qqaq=dirpe0xb9q8qiLsFr0=vr0=vr0dc8meaabaqaciaacaGaaeqabaWaaeGaeaaakeaaimaacqWFaeFqaaa@3821@. (size *k*) is generated according to an order *m *≥ 1 homogeneous, stationary and ergodic Markov model. Let *π *(size *k*^*m*+1^) defined by

*π*(*w*, *a*) = ℙ(*X*_*m*+1 _= *a*|*X*_1_...*X*_*m *_= *w*)     ∀(*w*, *a*) ∈ A
 MathType@MTEF@5@5@+=feaafiart1ev1aaatCvAUfKttLearuWrP9MDH5MBPbIqV92AaeXatLxBI9gBamrtHrhAL1wy0L2yHvtyaeHbnfgDOvwBHrxAJfwnaebbnrfifHhDYfgasaacH8akY=wiFfYdH8Gipec8Eeeu0xXdbba9frFj0=OqFfea0dXdd9vqai=hGuQ8kuc9pgc9s8qqaq=dirpe0xb9q8qiLsFr0=vr0=vr0dc8meaabaqaciaacaGaaeqabaWaaeGaeaaakeaaimaacqWFaeFqaaa@3821@^*m *^× A
 MathType@MTEF@5@5@+=feaafiart1ev1aaatCvAUfKttLearuWrP9MDH5MBPbIqV92AaeXatLxBI9gBamrtHrhAL1wy0L2yHvtyaeHbnfgDOvwBHrxAJfwnaebbnrfifHhDYfgasaacH8akY=wiFfYdH8Gipec8Eeeu0xXdbba9frFj0=OqFfea0dXdd9vqai=hGuQ8kuc9pgc9s8qqaq=dirpe0xb9q8qiLsFr0=vr0=vr0dc8meaabaqaciaacaGaaeqabaWaaeGaeaaakeaaimaacqWFaeFqaaa@3821@     (1)

be the parameter of this Markov model, Π its transition matrix (note that we have Π = *π *only if *m *= 1) and *μ *its stationary distribution (defined by *μ *× Π = *μ*).

We then introduce the pattern statistic defined by

S={−log⁡10ℙ(N≥Nobs)if Nobs≥E[N]log⁡10ℙ(N≤Nobs)if Nobs<E[N]     (2)
 MathType@MTEF@5@5@+=feaafiart1ev1aaatCvAUfKttLearuWrP9MDH5MBPbIqV92AaeXatLxBI9gBaebbnrfifHhDYfgasaacH8akY=wiFfYdH8Gipec8Eeeu0xXdbba9frFj0=OqFfea0dXdd9vqai=hGuQ8kuc9pgc9s8qqaq=dirpe0xb9q8qiLsFr0=vr0=vr0dc8meaabaqaciaacaGaaeqabaqabeGadaaakeaacqWGtbWucqGH9aqpdaGabeqaauaabiqaciaaaeaacqGHsislcyGGSbaBcqGGVbWBcqGGNbWzdaWgaaWcbaGaeGymaeJaeGimaadabeaatuuDJXwAK1uy0HMmaeHbfv3ySLgzG0uy0HgiuD3BaGabaOGae8xgHaLaeiikaGIaemOta4KaeyyzImRaemOta40aaSbaaSqaaiabb+gaVjabbkgaIjabbohaZbqabaGccqGGPaqkaeaacqqGPbqAcqqGMbGzcqqGGaaicqWGobGtdaWgaaWcbaGaee4Ba8MaeeOyaiMaee4CamhabeaakiabgwMiZkab=ri8fjabcUfaBjabd6eaojabc2faDbqaaiGbcYgaSjabc+gaVjabcEgaNnaaBaaaleaacqaIXaqmcqaIWaamaeqaaOGae8xgHaLaeiikaGIaemOta4KaeyizImQaemOta40aaSbaaSqaaiabb+gaVjabbkgaIjabbohaZbqabaGccqGGPaqkaeaacqqGPbqAcqqGMbGzcqqGGaaicqWGobGtdaWgaaWcbaGaee4Ba8MaeeOyaiMaee4CamhabeaakiabgYda8iab=ri8fjabcUfaBjabd6eaojabc2faDbaaaiaawUhaaiaaxMaacaWLjaWaaeWaaeaacqaIYaGmaiaawIcacaGLPaaaaaa@815C@

where *N *is the random number of overlapping occurrences (*i. e*. *X *= aababaaba contains three overlapping occurrences of aba but only two non-overlapping ones) of a given fixed pattern on the random sequence *X *and *N*_obs _is an observation.

When *π *is known (and hence *μ*), several statistical methods are available to compute *S*: exact computations [1-4], Gaussian [5,6], binomial [7,8], compound Poisson [9-11] or large deviations approximations [12]. But in general, the parameter *π *is not available and must be estimated. Let us denote by **N**_0 _(resp. **N**_1_) the (overlap) frequencies of all words of size *m *(resp. *m *+ 1) in the sequence *Y *= *Y*_1 _... *Y*_*n*_, then the Maximum-Likelihood Estimator (MLE) of *π *is given by

π^(w,a)=N1(wa)∑b∈AN1(wb)∀(w,a)∈Am×A     (3)
 MathType@MTEF@5@5@+=feaafiart1ev1aaatCvAUfKttLearuWrP9MDH5MBPbIqV92AaeXatLxBI9gBamrtHrhAL1wy0L2yHvtyaeHbnfgDOvwBHrxAJfwnaebbnrfifHhDYfgasaacH8akY=wiFfYdH8Gipec8Eeeu0xXdbba9frFj0=OqFfea0dXdd9vqai=hGuQ8kuc9pgc9s8qqaq=dirpe0xb9q8qiLsFr0=vr0=vr0dc8meaabaqaciaacaGaaeqabaWaaeGaeaaakeaafaqabeqacaaabaacciGaf8hWdaNbaKaacqGGOaakcqWG3bWDcqGGSaalcqWGHbqycqGGPaqkcqGH9aqpdaWcaaqaaGqabiab+5eaonaaBaaaleaacqaIXaqmaeqaaOGaeiikaGIaem4DaCNaemyyaeMaeiykaKcabaWaaabeaeaacqGFobGtdaWgaaWcbaGaeGymaedabeaakiabcIcaOiabdEha3jabdkgaIjabcMcaPaWcbaGaemOyaiMaeyicI4mcdaGae0haXheabeqdcqGHris5aaaaaOqaaiabgcGiIiabcIcaOiabdEha3jabcYcaSiabdggaHjabcMcaPiabgIGiolab9bq8bnaaCaaaleqabaGaemyBa0gaaOGaey41aqRae0haXheaaiaaxMaacaWLjaGaeiikaGIaeG4mamJaeiykaKcaaa@6555@

and the MLE of *μ *(as a function of *π*) is therefore defined by μ^
 MathType@MTEF@5@5@+=feaafiart1ev1aaatCvAUfKttLearuWrP9MDH5MBPbIqV92AaeXatLxBI9gBaebbnrfifHhDYfgasaacH8akY=wiFfYdH8Gipec8Eeeu0xXdbba9frFj0=OqFfea0dXdd9vqai=hGuQ8kuc9pgc9s8qqaq=dirpe0xb9q8qiLsFr0=vr0=vr0dc8meaabaqaciaacaGaaeqabaqabeGadaaakeaaiiGacuWF8oqBgaqcaaaa@2E79@ × Π^
 MathType@MTEF@5@5@+=feaafiart1ev1aaatCvAUfKttLearuWrP9MDH5MBPbIqV92AaeXatLxBI9gBaebbnrfifHhDYfgasaacH8akY=wiFfYdH8Gipec8Eeeu0xXdbba9frFj0=OqFfea0dXdd9vqai=hGuQ8kuc9pgc9s8qqaq=dirpe0xb9q8qiLsFr0=vr0=vr0dc8meaabaqaciaacaGaaeqabaqabeGadaaakeaacuqHGoaugaqcaaaa@2E3A@ = μ^
 MathType@MTEF@5@5@+=feaafiart1ev1aaatCvAUfKttLearuWrP9MDH5MBPbIqV92AaeXatLxBI9gBaebbnrfifHhDYfgasaacH8akY=wiFfYdH8Gipec8Eeeu0xXdbba9frFj0=OqFfea0dXdd9vqai=hGuQ8kuc9pgc9s8qqaq=dirpe0xb9q8qiLsFr0=vr0=vr0dc8meaabaqaciaacaGaaeqabaqabeGadaaakeaaiiGacuWF8oqBgaqcaaaa@2E79@ where Π^
 MathType@MTEF@5@5@+=feaafiart1ev1aaatCvAUfKttLearuWrP9MDH5MBPbIqV92AaeXatLxBI9gBaebbnrfifHhDYfgasaacH8akY=wiFfYdH8Gipec8Eeeu0xXdbba9frFj0=OqFfea0dXdd9vqai=hGuQ8kuc9pgc9s8qqaq=dirpe0xb9q8qiLsFr0=vr0=vr0dc8meaabaqaciaacaGaaeqabaqabeGadaaakeaacuqHGoaugaqcaaaa@2E3A@ is the transition matrix associated to π^
 MathType@MTEF@5@5@+=feaafiart1ev1aaatCvAUfKttLearuWrP9MDH5MBPbIqV92AaeXatLxBI9gBaebbnrfifHhDYfgasaacH8akY=wiFfYdH8Gipec8Eeeu0xXdbba9frFj0=OqFfea0dXdd9vqai=hGuQ8kuc9pgc9s8qqaq=dirpe0xb9q8qiLsFr0=vr0=vr0dc8meaabaqaciaacaGaaeqabaqabeGadaaakeaaiiGacuWFapaCgaqcaaaa@2E80@

We introduce now the following estimators

μN(w)=N0(w)n−m+1andπN(w,a)=N1(wa)N0(w)∀(w,a)∈Am×A     (4)
 MathType@MTEF@5@5@+=feaafiart1ev1aaatCvAUfKttLearuWrP9MDH5MBPbIqV92AaeXatLxBI9gBamrtHrhAL1wy0L2yHvtyaeHbnfgDOvwBHrxAJfwnaebbnrfifHhDYfgasaacH8akY=wiFfYdH8Gipec8Eeeu0xXdbba9frFj0=OqFfea0dXdd9vqai=hGuQ8kuc9pgc9s8qqaq=dirpe0xb9q8qiLsFr0=vr0=vr0dc8meaabaqaciaacaGaaeqabaWaaeGaeaaakeaafaqabeqaeaaaaeaaiiGacqWF8oqBdaWgaaWcbaacbeGae4Nta4eabeaakiabcIcaOiabdEha3jabcMcaPiabg2da9maalaaabaGae4Nta40aaSbaaSqaaiabicdaWaqabaGccqGGOaakcqWG3bWDcqGGPaqkaeaacqWGUbGBcqGHsislcqWGTbqBcqGHRaWkcqaIXaqmaaaabaacbaGae0xyaeMae0NBa4Mae0hzaqgabaGae8hWda3aaSbaaSqaaiab+5eaobqabaGccqGGOaakcqWG3bWDcqGGSaalcqWGHbqycqGGPaqkcqGH9aqpdaWcaaqaaiab+5eaonaaBaaaleaacqaIXaqmaeqaaOGaeiikaGIaem4DaCNaemyyaeMaeiykaKcabaGae4Nta40aaSbaaSqaaiabicdaWaqabaGccqGGOaakcqWG3bWDcqGGPaqkaaaabaGaeyiaIiIaeiikaGIaem4DaCNaeiilaWIaemyyaeMaeiykaKIaeyicI4mcdaGaeWhaXh0aaWbaaSqabeaacqWGTbqBaaGccqGHxdaTaaGaeWhaXhKaaCzcaiaaxMaacqGGOaakcqaI0aancqGGPaqkaaa@750F@

which are known to be asymptotically equivalent with the MLE when *n *is large.

The quality of parameter estimation depends both on the number of parameters to estimate (*k*^*m*+1 ^for an order *m *Markov model) and of the length (*n*) of the homogeneous sequence used for their estimation. When the same sequence (or set of sequences) is used both for observed frequencies and parameter estimation, *m *should not be greater than *h *– 2 for a pattern of length *h *(as else, the observed frequency of the pattern will be included in the model). As literature often suggests to use the highest possible order, it is hence common to consider *m *= 6 or more (for a DNA pattern of size *h *≥ 8). Moreover, because of the homogeneity assumption of the model, the considered genomes have often to be segmented first. As a result, the sequences length used for parameter estimations are often dramatically reduced by such segmentation (*e. g*. *n *= 10^5 ^to *n *= 10^6 ^at the very best for DNA sequences). It is hence quite common to encounter high order Markov models estimated on rather short sequences which could result in high sensitivity to parameter estimation.

Considering that *Y *is generated through a Markov model of parameter *π*, the main goal of this paper is to study the distribution of *S*_**N**_, the statistic *S *computed using the estimators *μ*_**N **_and *π*_**N**_, and the consequences of its variability in projects using pattern statistics. We first present in details how the delta-method can be used to get a Gaussian approximation for the distribution of *S*_**N **_(using a binomial approximation to compute the pattern statistics). Then these approximations are validated through simulations and, at last, we consider a classical pattern study (finding the most over-represented patterns of a given size) and we evaluate the detrimental effect of parameter estimations both in terms of true positive rate and rank accordance.

## Materials and methods

### Distribution of N = (N_0_, N_1_)

As the estimators defined in (4) are expressed as functions of **N**_0 _and **N**_1 _we first study their distribution. Using a Gaussian approximation, we have

ℒ([N0N1]︸N)≃N([E0E1]︸E,[C0,0C0,1C1,0C1,1]︸C)     (5)
 MathType@MTEF@5@5@+=feaafiart1ev1aaatCvAUfKttLearuWrP9MDH5MBPbIqV92AaeXatLxBI9gBamrtHrhAL1wy0L2yHvtyaeHbnfgDOvwBHrxAJfwnaebbnrfifHhDYfgasaacH8akY=wiFfYdH8Gipec8Eeeu0xXdbba9frFj0=OqFfea0dXdd9vqai=hGuQ8kuc9pgc9s8qqaq=dirpe0xb9q8qiLsFr0=vr0=vr0dc8meaabaqaciaacaGaaeqabaWaaeGaeaaakeaaimaacqWFsectdaqadaqaamaayaaabaWaamWaaeaafaqabeGabaaabaacbeGae4Nta40aaSbaaSqaaiabicdaWaqabaaakeaacqGFobGtdaWgaaWcbaGaeGymaedabeaaaaaakiaawUfacaGLDbaaaSqaaiab+5eaobGccaGL44paaiaawIcacaGLPaaacqWIdjYocqWFneVtdaqadaqaamaayaaabaWaamWaaeaafaqabeGabaaabaGae4xrau0aaSbaaSqaaiabicdaWaqabaaakeaacqGFfbqrdaWgaaWcbaGaeGymaedabeaaaaaakiaawUfacaGLDbaaaSqaaiab+veafbGccaGL44pacqGGSaaldaagaaqaamaadmaabaqbaeqabiGaaaqaaiab+neadnaaBaaaleaacqaIWaamcqGGSaalcqaIWaamaeqaaaGcbaGae43qam0aaSbaaSqaaiabicdaWiabcYcaSiabigdaXaqabaaakeaacqGFdbWqdaWgaaWcbaGaeGymaeJaeiilaWIaeGimaadabeaaaOqaaiab+neadnaaBaaaleaacqaIXaqmcqGGSaalcqaIXaqmaeqaaaaaaOGaay5waiaaw2faaaWcbaGae43qameakiaawIJ=aaGaayjkaiaawMcaaiaaxMaacaWLjaGaeiikaGIaeGynauJaeiykaKcaaa@6A7A@

where, for *i*, *j *∈ {0, 1}, **E**_*i *_∈ ℝdi
 MathType@MTEF@5@5@+=feaafiart1ev1aaatCvAUfKttLearuWrP9MDH5MBPbIqV92AaeXatLxBI9gBaebbnrfifHhDYfgasaacH8akY=wiFfYdH8Gipec8Eeeu0xXdbba9frFj0=OqFfea0dXdd9vqai=hGuQ8kuc9pgc9s8qqaq=dirpe0xb9q8qiLsFr0=vr0=vr0dc8meaabaqaciaacaGaaeqabaqabeGadaaakeaacqWIDesOdaahaaWcbeqaaiabdsgaKnaaBaaameaacqWGPbqAaeqaaaaaaaa@3122@, and **C**_*i,j *_∈ ℝdi
 MathType@MTEF@5@5@+=feaafiart1ev1aaatCvAUfKttLearuWrP9MDH5MBPbIqV92AaeXatLxBI9gBaebbnrfifHhDYfgasaacH8akY=wiFfYdH8Gipec8Eeeu0xXdbba9frFj0=OqFfea0dXdd9vqai=hGuQ8kuc9pgc9s8qqaq=dirpe0xb9q8qiLsFr0=vr0=vr0dc8meaabaqaciaacaGaaeqabaqabeGadaaakeaacqWIDesOdaahaaWcbeqaaiabdsgaKnaaBaaameaacqWGPbqAaeqaaaaaaaa@3122@ × ℝdj
 MathType@MTEF@5@5@+=feaafiart1ev1aaatCvAUfKttLearuWrP9MDH5MBPbIqV92AaeXatLxBI9gBaebbnrfifHhDYfgasaacH8akY=wiFfYdH8Gipec8Eeeu0xXdbba9frFj0=OqFfea0dXdd9vqai=hGuQ8kuc9pgc9s8qqaq=dirpe0xb9q8qiLsFr0=vr0=vr0dc8meaabaqaciaacaGaaeqabaqabeGadaaakeaacqWIDesOdaahaaWcbeqaaiabdsgaKnaaBaaameaacqWGQbGAaeqaaaaaaaa@3124@ with *d*_*i *_= *k*^*m*+*i*^. One can note that **C**_0,0 _and **C**_1,1 _are symmetric, and ^*t *^(**C**_1,0_) = **C**_0,1 _(where ^*t *^is the matrix transpose operator).

In the stationary case, exact expression of **E **and **C **can be computed according to [5].

Expectation is simply given ∀*w *∈ A
 MathType@MTEF@5@5@+=feaafiart1ev1aaatCvAUfKttLearuWrP9MDH5MBPbIqV92AaeXatLxBI9gBamrtHrhAL1wy0L2yHvtyaeHbnfgDOvwBHrxAJfwnaebbnrfifHhDYfgasaacH8akY=wiFfYdH8Gipec8Eeeu0xXdbba9frFj0=OqFfea0dXdd9vqai=hGuQ8kuc9pgc9s8qqaq=dirpe0xb9q8qiLsFr0=vr0=vr0dc8meaabaqaciaacaGaaeqabaWaaeGaeaaakeaaimaacqWFaeFqaaa@3821@^*m *^by

**E**_0_(*w*) = (*n *- *m *+ 1) *μ*(*w*)     **E**_1 _(*wa*) = (*n *- *m*) *μ*(*w*)Π(*w*, *a*)     ∀(*w*, *a*) ∈ A
 MathType@MTEF@5@5@+=feaafiart1ev1aaatCvAUfKttLearuWrP9MDH5MBPbIqV92AaeXatLxBI9gBamrtHrhAL1wy0L2yHvtyaeHbnfgDOvwBHrxAJfwnaebbnrfifHhDYfgasaacH8akY=wiFfYdH8Gipec8Eeeu0xXdbba9frFj0=OqFfea0dXdd9vqai=hGuQ8kuc9pgc9s8qqaq=dirpe0xb9q8qiLsFr0=vr0=vr0dc8meaabaqaciaacaGaaeqabaWaaeGaeaaakeaaimaacqWFaeFqaaa@3821@^*m *^× A
 MathType@MTEF@5@5@+=feaafiart1ev1aaatCvAUfKttLearuWrP9MDH5MBPbIqV92AaeXatLxBI9gBamrtHrhAL1wy0L2yHvtyaeHbnfgDOvwBHrxAJfwnaebbnrfifHhDYfgasaacH8akY=wiFfYdH8Gipec8Eeeu0xXdbba9frFj0=OqFfea0dXdd9vqai=hGuQ8kuc9pgc9s8qqaq=dirpe0xb9q8qiLsFr0=vr0=vr0dc8meaabaqaciaacaGaaeqabaWaaeGaeaaakeaaimaacqWFaeFqaaa@3821@     (6)

In order to give more fluidity to this paper, the expression of the covariance matrix **C **have been moved in appendix A. Let us remark, before going forward that substituting **N **by **E **in (4) immediately gives

μE=μandπE=(1−1n−m+1)π     (7)
 MathType@MTEF@5@5@+=feaafiart1ev1aaatCvAUfKttLearuWrP9MDH5MBPbIqV92AaeXatLxBI9gBaebbnrfifHhDYfgasaacH8akY=wiFfYdH8Gipec8Eeeu0xXdbba9frFj0=OqFfea0dXdd9vqai=hGuQ8kuc9pgc9s8qqaq=dirpe0xb9q8qiLsFr0=vr0=vr0dc8meaabaqaciaacaGaaeqabaqabeGadaaakeaafaqabeqadaaabaacciGae8hVd02aaSbaaSqaaGqabiab+veafbqabaGccqGH9aqpcqWF8oqBaeaacqqGHbqycqqGUbGBcqqGKbazaeaacqWFapaCdaWgaaWcbaGae4xraueabeaakiabg2da9maabmaabaGaeGymaeJaeyOeI0YaaSaaaeaacqaIXaqmaeaacqWGUbGBcqGHsislcqWGTbqBcqGHRaWkcqaIXaqmaaaacaGLOaGaayzkaaGae8hWdaNaaCzcaiaaxMaadaqadaqaaiabiEda3aGaayjkaiaawMcaaaaaaaa@49E8@

### Delta method

Let us start with a simple case. We consider a single pattern which is over-represented (seen more than expected) so we have

SN=−log⁡10F+(N)withF+(N)≜ℙμN,πN(N≥Nobs)     (8)
 MathType@MTEF@5@5@+=feaafiart1ev1aaatCvAUfKttLearuWrP9MDH5MBPbIqV92AaeXatLxBI9gBaebbnrfifHhDYfgasaacH8akY=wiFfYdH8Gipec8Eeeu0xXdbba9frFj0=OqFfea0dXdd9vqai=hGuQ8kuc9pgc9s8qqaq=dirpe0xb9q8qiLsFr0=vr0=vr0dc8meaabaqaciaacaGaaeqabaqabeGadaaakeaafaqaaeqadaaabaGaem4uam1aaSbaaSqaaGqabiab=5eaobqabaGccqGH9aqpcqGHsislcyGGSbaBcqGGVbWBcqGGNbWzdaWgaaWcbaGaeGymaeJaeGimaadabeaakiabdAeagnaaCaaaleqabaGaey4kaScaaOGaeiikaGIae8Nta4KaeiykaKcabaGaee4DaCNaeeyAaKMaeeiDaqNaeeiAaGgabaGaemOray0aaWbaaSqabeaacqGHRaWkaaGccqGGOaakcqWFobGtcqGGPaqkcqWICjcqtuuDJXwAK1uy0HMmaeHbfv3ySLgzG0uy0HgiuD3BaGabaiab+LriqnaaBaaaleaaiiGacqqF8oqBdaWgaaadbaGae8Nta4eabeaaliabcYcaSiab9b8aWnaaBaaameaacqWFobGtaeqaaaWcbeaakiabcIcaOiabd6eaojabgwMiZkabd6eaonaaBaaaleaacqqGVbWBcqqGIbGycqqGZbWCaeqaaOGaeiykaKcaaiaaxMaacaWLjaWaaeWaaeaacqaI4aaoaiaawIcacaGLPaaaaaa@681D@

where the function *F*^+ ^also depends on the sequence length ℓ and the considered pattern.

If *F*^+ ^is differentiate, the delta-method (a simple first order Taylor expansion around **N **= **E**, see [13]) provides the following approximation:

SN≃−log⁡10F+(E)− t(N−E)∇F+(E)ln⁡(10)F+(E)     (9)
 MathType@MTEF@5@5@+=feaafiart1ev1aaatCvAUfKttLearuWrP9MDH5MBPbIqV92AaeXatLxBI9gBaebbnrfifHhDYfgasaacH8akY=wiFfYdH8Gipec8Eeeu0xXdbba9frFj0=OqFfea0dXdd9vqai=hGuQ8kuc9pgc9s8qqaq=dirpe0xb9q8qiLsFr0=vr0=vr0dc8meaabaqaciaacaGaaeqabaqabeGadaaakeaacqWGtbWudaWgaaWcbaacbeGae8Nta4eabeaakiabloKi7iabgkHiTiGbcYgaSjabc+gaVjabcEgaNnaaBaaaleaacqaIXaqmcqaIWaamaeqaaOGaemOray0aaWbaaSqabeaacqGHRaWkaaGccqGGOaakcqWFfbqrcqGGPaqkcqGHsisldaWcaaqaaiabbccaGmaaCaaaleqabaGaemiDaqhaaOGaeiikaGIae8Nta4KaeyOeI0Iae8xrauKaeiykaKIaey4bIeTaemOray0aaWbaaSqabeaacqGHRaWkaaGccqGGOaakcqWFfbqrcqGGPaqkaeaacyGGSbaBcqGGUbGBcqGGOaakcqaIXaqmcqaIWaamcqGGPaqkcqWGgbGrdaahaaWcbeqaaiabgUcaRaaakiabcIcaOiab=veafjabcMcaPaaacaWLjaGaaCzcamaabmaabaGaeGyoaKdacaGLOaGaayzkaaaaaa@5A3D@

and hence, using (7) we have

SN≃S− t(N−E)∇F+(E)ln⁡(10)F+(E)     (10)
 MathType@MTEF@5@5@+=feaafiart1ev1aaatCvAUfKttLearuWrP9MDH5MBPbIqV92AaeXatLxBI9gBaebbnrfifHhDYfgasaacH8akY=wiFfYdH8Gipec8Eeeu0xXdbba9frFj0=OqFfea0dXdd9vqai=hGuQ8kuc9pgc9s8qqaq=dirpe0xb9q8qiLsFr0=vr0=vr0dc8meaabaqaciaacaGaaeqabaqabeGadaaakeaacqWGtbWudaWgaaWcbaacbeGae8Nta4eabeaakiabloKi7iabdofatjabgkHiTmaalaaabaGaeeiiaaYaaWbaaSqabeaacqWG0baDaaGccqGGOaakcqWFobGtcqGHsislcqWFfbqrcqGGPaqkcqGHhis0cqWGgbGrdaahaaWcbeqaaiabgUcaRaaakiabcIcaOiab=veafjabcMcaPaqaaiGbcYgaSjabc6gaUjabcIcaOiabigdaXiabicdaWiabcMcaPiabdAeagnaaCaaaleqabaGaey4kaScaaOGaeiikaGIae8xrauKaeiykaKcaaiaaxMaacaWLjaWaaeWaaeaacqaIXaqmcqaIWaamaiaawIcacaGLPaaaaaa@503C@

for *n *large enough. The distribution of S^
 MathType@MTEF@5@5@+=feaafiart1ev1aaatCvAUfKttLearuWrP9MDH5MBPbIqV92AaeXatLxBI9gBaebbnrfifHhDYfgasaacH8akY=wiFfYdH8Gipec8Eeeu0xXdbba9frFj0=OqFfea0dXdd9vqai=hGuQ8kuc9pgc9s8qqaq=dirpe0xb9q8qiLsFr0=vr0=vr0dc8meaabaqaciaacaGaaeqabaqabeGadaaakeaacuWGtbWugaqcaaaa@2DEB@ is therefore approximated by

L
 MathType@MTEF@5@5@+=feaafiart1ev1aaatCvAUfKttLearuWrP9MDH5MBPbIqV92AaeXatLxBI9gBaebbnrfifHhDYfgasaacH8akY=wiFfYdH8Gipec8Eeeu0xXdbba9frFj0=OqFfea0dXdd9vqai=hGuQ8kuc9pgc9s8qqaq=dirpe0xb9q8qiLsFr0=vr0=vr0dc8meaabaqaciaacaGaaeqabaqabeGadaaakeaatCvAUfeBSjuyZL2yd9gzLbvyNv2CaeHbnf2C0vMCJfMCKbaceiGaa8htaaaa@394B@ (*S*_**N**_) ≃ N
 MathType@MTEF@5@5@+=feaafiart1ev1aaatCvAUfKttLearuWrP9MDH5MBPbIqV92AaeXatLxBI9gBamrtHrhAL1wy0L2yHvtyaeHbnfgDOvwBHrxAJfwnaebbnrfifHhDYfgasaacH8akY=wiFfYdH8Gipec8Eeeu0xXdbba9frFj0=OqFfea0dXdd9vqai=hGuQ8kuc9pgc9s8qqaq=dirpe0xb9q8qiLsFr0=vr0=vr0dc8meaabaqaciaacaGaaeqabaWaaeGaeaaakeaaimaacqWFneVtaaa@383B@ (*S*, *σ*^2^)     (11)

with

σ= t∇F+(E)×C×∇F+(E)ln⁡(10)F+(E)     (12)
 MathType@MTEF@5@5@+=feaafiart1ev1aaatCvAUfKttLearuWrP9MDH5MBPbIqV92AaeXatLxBI9gBaebbnrfifHhDYfgasaacH8akY=wiFfYdH8Gipec8Eeeu0xXdbba9frFj0=OqFfea0dXdd9vqai=hGuQ8kuc9pgc9s8qqaq=dirpe0xb9q8qiLsFr0=vr0=vr0dc8meaabaqaciaacaGaaeqabaqabeGadaaakeaaiiGacqWFdpWCcqGH9aqpdaWcaaqaamaakaaabaGaeeiiaaYaaWbaaSqabeaacqWG0baDaaGccqGHhis0cqWGgbGrdaahaaWcbeqaaiabgUcaRaaakiabcIcaOGqabiab+veafjabcMcaPiabgEna0kab+neadjabgEna0kabgEGirlabdAeagnaaCaaaleqabaGaey4kaScaaOGaeiikaGIae4xrauKaeiykaKcaleqaaaGcbaGagiiBaWMaeiOBa4MaeiikaGIaeGymaeJaeGimaaJaeiykaKIaemOray0aaWbaaSqabeaacqGHRaWkaaGccqGGOaakcqGFfbqrcqGGPaqkaaGaaCzcaiaaxMaadaqadaqaaiabigdaXiabikdaYaGaayjkaiaawMcaaaaa@543D@

In consequence, computing *σ *requires both to compute **C **(done in appendix A) and ∇*F*^+ ^(**E**).

### Single pattern

The exact expression of *F*^+ ^is computable through many different methods [1-4] but is too much complicated to derive explicitly ∇*F*^+^. To overcome this problem, we propose to consider an approximation of *F*^+^. As said in introduction, many kind of approximations are available (Gaussian, binomial, compound Poisson or large deviations). In this paper, we have chosen to use a binomial approximation as it provides an expression which is analytically differentiable and is known to be a good heuristic to the problem [8].

For a single non-degenerate pattern (*i.e*. a simple word) *W *= *w*_1 _... *w*_*h *_(*w*_*i *_∈ A
 MathType@MTEF@5@5@+=feaafiart1ev1aaatCvAUfKttLearuWrP9MDH5MBPbIqV92AaeXatLxBI9gBamrtHrhAL1wy0L2yHvtyaeHbnfgDOvwBHrxAJfwnaebbnrfifHhDYfgasaacH8akY=wiFfYdH8Gipec8Eeeu0xXdbba9frFj0=OqFfea0dXdd9vqai=hGuQ8kuc9pgc9s8qqaq=dirpe0xb9q8qiLsFr0=vr0=vr0dc8meaabaqaciaacaGaaeqabaWaaeGaeaaakeaaimaacqWFaeFqaaa@3821@) with *h *≥ *m *- 1 we first denote by

*P*(**N**) = *μ*_**N **_(*w*_1 _... *w*_*m*_) × *π*_**N **_(*w*_1 _... *w*_*m*_, *w*_*m*+1_) × ... × *π*_**N **_(*w*_*h*-*m *_... *w*_*h*-1_, *w*_*h*_)     (13)

the probability for *W *to occur at a given position in the sequence and then we get

F+(N)≃ℙ(ℬ(ℓh,P(N))≥Nobs)=β(P(N),Nobs,ℓh−Nobs+1)β(Nobs,ℓh−Nobs+1)     (14)
 MathType@MTEF@5@5@+=feaafiart1ev1aaatCvAUfKttLearuWrP9MDH5MBPbIqV92AaeXatLxBI9gBamrtHrhAL1wy0L2yHvtyaeHbnfgDOvwBHrxAJfwnaebbnrfifHhDYfgasaacH8akY=wiFfYdH8Gipec8Eeeu0xXdbba9frFj0=OqFfea0dXdd9vqai=hGuQ8kuc9pgc9s8qqaq=dirpe0xb9q8qiLsFr0=vr0=vr0dc8meaabaqaciaacaGaaeqabaWaaeGaeaaakeaacqWGgbGrdaahaaWcbeqaaiabgUcaRaaakiabcIcaOGqabiab=5eaojabcMcaPiabloKi7mrr1ngBPrwtHrhAYaqehuuDJXwAKbstHrhAGq1DVbacfaGae4xgHaLaeiikaGccdaGae0hlHiKaeiikaGIaeS4eHW2aaSbaaSqaaiabdIgaObqabaGccqGGSaalcqWGqbaucqGGOaakcqWFobGtcqGGPaqkcqGGPaqkcqGHLjYScqWGobGtdaWgaaWcbaacbaGaeW3Ba8MaeWNyaiMaeW3CamhabeaakiabcMcaPiabg2da9maalaaabaacciGaeSNSdiMaeiikaGIaemiuaaLaeiikaGIae8Nta4KaeiykaKIaeiilaWIaemOta40aaSbaaSqaaiabb+gaVjabbkgaIjabbohaZbqabaGccqGGSaalcqWItecBdaWgaaWcbaGaemiAaGgabeaakiabgkHiTiabd6eaonaaBaaaleaacqaFVbWBcqaFIbGycqaFZbWCaeqaaOGaey4kaSIaeGymaeJaeiykaKcabaGaeSNSdiMaeiikaGIaemOta40aaSbaaSqaaiab89gaVjab8jgaIjab8nhaZbqabaGccqGGSaalcqWItecBdaWgaaWcbaGaemiAaGgabeaakiabgkHiTiabd6eaonaaBaaaleaacqaFVbWBcqaFIbGycqaFZbWCaeqaaOGaey4kaSIaeGymaeJaeiykaKcaaiaaxMaacaWLjaGaeiikaGIaeGymaeJaeGinaqJaeiykaKcaaa@8E81@

where ℬ
 MathType@MTEF@5@5@+=feaafiart1ev1aaatCvAUfKttLearuWrP9MDH5MBPbIqV92AaeXatLxBI9gBamrtHrhAL1wy0L2yHvtyaeHbnfgDOvwBHrxAJfwnaebbnrfifHhDYfgasaacH8akY=wiFfYdH8Gipec8Eeeu0xXdbba9frFj0=OqFfea0dXdd9vqai=hGuQ8kuc9pgc9s8qqaq=dirpe0xb9q8qiLsFr0=vr0=vr0dc8meaabaqaciaacaGaaeqabaWaaeGaeaaakeaaimaacqWFSeIqaaa@377E@ denotes the binomial distribution, with ℓ_*h *_= ℓ - *h *+ 1 and where the β functions (complete and incomplete) and their relation to the binomial cumulative distribution function are described in appendix B.

Note that if we consider non-overlapping occurrences instead of overlapping ones, we can still use a binomial approximation for the distribution of *N*, but the expression of *P*(**N**) is more complicated as it involves the auto-correlation polynome of the pattern [14]. This point is not developed in this paper.

Replacing *μ*_**N **_and *π*_**N **_by their expression easily gives

P(N)=1n−m+1∏w∈Am∏a∈AN1(wa)A1(wa)N0(w)A0(w)     (15)
MathType@MTEF@5@5@+=feaafiart1ev1aaatCvAUfKttLearuWrP9MDH5MBPbIqV92AaeXatLxBI9gBamrtHrhAL1wy0L2yHvtyaeHbnfgDOvwBHrxAJfwnaebbnrfifHhDYfgasaacH8akY=wiFfYdH8Gipec8Eeeu0xXdbba9frFj0=OqFfea0dXdd9vqai=hGuQ8kuc9pgc9s8qqaq=dirpe0xb9q8qiLsFr0=vr0=vr0dc8meaabaqaciaacaGaaeqabaWaaeGaeaaakeaacqWGqbaucqGGOaakieqacqWFobGtcqGGPaqkcqGH9aqpdaWcaaqaaiabigdaXaqaaiabd6gaUjabgkHiTiabd2gaTjabgUcaRiabigdaXaaadaqeqbqaamaalaaabaWaaebeaeaacqWFobGtdaWgaaWcbaGaeGymaedabeaakiabcIcaOiabdEha3jabdggaHjabcMcaPmaaCaaaleqabaGaemyqae0aaSbaaWqaaiabigdaXaqabaWccqGGOaakcqWG3bWDcqWGHbqycqGGPaqkaaaabaGaemyyaeMaeyicI4mcdaGae4haXheabeqdcqGHpis1aaGcbaGae8Nta40aaSbaaSqaaiabicdaWaqabaGccqGGOaakcqWG3bWDcqGGPaqkdaahaaWcbeqaaiabdgeabnaaBaaameaacqaIWaamaeqaaSGaeiikaGIaem4DaCNaeiykaKcaaaaaaeaacqWG3bWDcqGHiiIZcqGFaeFqdaahaaadbeqaaiabd2gaTbaaaSqab0Gaey4dIunakiaaxMaacaWLjaGaeiikaGIaeGymaeJaeGynauJaeiykaKcaaa@6E23@

where *A*_1_(*wa*) counts occurrences of the word *wa *in *W *= *w*_1 _... *w*_*h *_and *A*_0 _(*w*) counts occurrences of the word *w *in *w*_2 _... *w*_*h*-1_. Note that in the particular case where *h *= *m *- 1, all *A*_0 _(*w*) are null and we simply get (*n *- *m *+ l) × *P *(**N**) = **N**_1 _(*W*).

Using the derivative properties of the incomplete beta function (see appendix B for more details) we hence get

∇F+(N)≃P(N)Nobs−1(1−P(N))ℓh−Nobsβ(Nobs,ℓh−Nobs+1)×∇P(N)     (16)
 MathType@MTEF@5@5@+=feaafiart1ev1aaatCvAUfKttLearuWrP9MDH5MBPbIqV92AaeXatLxBI9gBaebbnrfifHhDYfgasaacH8akY=wiFfYdH8Gipec8Eeeu0xXdbba9frFj0=OqFfea0dXdd9vqai=hGuQ8kuc9pgc9s8qqaq=dirpe0xb9q8qiLsFr0=vr0=vr0dc8meaabaqaciaacaGaaeqabaqabeGadaaakeaacqGHhis0cqWGgbGrdaahaaWcbeqaaiabgUcaRaaakiabcIcaOGqabiab=5eaojabcMcaPiabloKi7maalaaabaGaemiuaaLaeiikaGIae8Nta4KaeiykaKYaaWbaaSqabeaacqWGobGtdaWgaaadbaGaee4Ba8MaeeOyaiMaee4CamhabeaaliabgkHiTiabigdaXaaakiabcIcaOiabigdaXiabgkHiTiabdcfaqjabcIcaOiab=5eaojabcMcaPiabcMcaPmaaCaaaleqabaGaeS4eHW2aaSbaaWqaaiabdIgaObqabaWccqGHsislcqWGobGtdaWgaaadbaGaee4Ba8MaeeOyaiMaee4CamhabeaaaaaakeaaiiGacqGFYoGycqGGOaakcqWGobGtdaWgaaWcbaGaee4Ba8MaeeOyaiMaee4CamhabeaakiabcYcaSiabloriSnaaBaaaleaacqWGObaAaeqaaOGaeyOeI0IaemOta40aaSbaaSqaaiabb+gaVjabbkgaIjabbohaZbqabaGccqGHRaWkcqaIXaqmcqGGPaqkaaGaey41aqRaey4bIeTaemiuaaLaeiikaGIae8Nta4KaeiykaKIaaCzcaiaaxMaadaqadaqaaiabigdaXiabiAda2aGaayjkaiaawMcaaaaa@71E0@

so all we need is to compute ∇*P*(**N**).

For all (*w*, *a*) ∈ A
 MathType@MTEF@5@5@+=feaafiart1ev1aaatCvAUfKttLearuWrP9MDH5MBPbIqV92AaeXatLxBI9gBamrtHrhAL1wy0L2yHvtyaeHbnfgDOvwBHrxAJfwnaebbnrfifHhDYfgasaacH8akY=wiFfYdH8Gipec8Eeeu0xXdbba9frFj0=OqFfea0dXdd9vqai=hGuQ8kuc9pgc9s8qqaq=dirpe0xb9q8qiLsFr0=vr0=vr0dc8meaabaqaciaacaGaaeqabaWaaeGaeaaakeaaimaacqWFaeFqaaa@3821@^*m *^× A
 MathType@MTEF@5@5@+=feaafiart1ev1aaatCvAUfKttLearuWrP9MDH5MBPbIqV92AaeXatLxBI9gBamrtHrhAL1wy0L2yHvtyaeHbnfgDOvwBHrxAJfwnaebbnrfifHhDYfgasaacH8akY=wiFfYdH8Gipec8Eeeu0xXdbba9frFj0=OqFfea0dXdd9vqai=hGuQ8kuc9pgc9s8qqaq=dirpe0xb9q8qiLsFr0=vr0=vr0dc8meaabaqaciaacaGaaeqabaWaaeGaeaaakeaaimaacqWFaeFqaaa@3821@ we have

∂P(N)∂N0(w)=−A0(w)N0(w)×P(N)     (17)
 MathType@MTEF@5@5@+=feaafiart1ev1aaatCvAUfKttLearuWrP9MDH5MBPbIqV92AaeXatLxBI9gBaebbnrfifHhDYfgasaacH8akY=wiFfYdH8Gipec8Eeeu0xXdbba9frFj0=OqFfea0dXdd9vqai=hGuQ8kuc9pgc9s8qqaq=dirpe0xb9q8qiLsFr0=vr0=vr0dc8meaabaqaciaacaGaaeqabaqabeGadaaakeaadaWcaaqaaiabgkGi2kabdcfaqjabcIcaOGqabiab=5eaojabcMcaPaqaaiabgkGi2kab=5eaonaaBaaaleaaieaacqGFWaamaeqaaOGaeiikaGIaem4DaCNaeiykaKcaaiabg2da9iabgkHiTmaalaaabaGaemyqae0aaSbaaSqaaiabicdaWaqabaGccqGGOaakcqWG3bWDcqGGPaqkaeaacqWFobGtdaWgaaWcbaGae4hmaadabeaakiabcIcaOiabdEha3jabcMcaPaaacqGHxdaTcqWGqbaucqGGOaakcqWFobGtcqGGPaqkcaWLjaGaaCzcamaabmaabaGaeGymaeJaeG4naCdacaGLOaGaayzkaaaaaa@5086@

and

∂P(N)∂N1(w)=−A1(wa)N1(wa)×P(N)     (18)
 MathType@MTEF@5@5@+=feaafiart1ev1aaatCvAUfKttLearuWrP9MDH5MBPbIqV92AaeXatLxBI9gBaebbnrfifHhDYfgasaacH8akY=wiFfYdH8Gipec8Eeeu0xXdbba9frFj0=OqFfea0dXdd9vqai=hGuQ8kuc9pgc9s8qqaq=dirpe0xb9q8qiLsFr0=vr0=vr0dc8meaabaqaciaacaGaaeqabaqabeGadaaakeaadaWcaaqaaiabgkGi2kabdcfaqjabcIcaOGqabiab=5eaojabcMcaPaqaaiabgkGi2kab=5eaonaaBaaaleaaieaacqGFXaqmaeqaaOGaeiikaGIaem4DaCNaeiykaKcaaiabg2da9iabgkHiTmaalaaabaGaemyqae0aaSbaaSqaaiabigdaXaqabaGccqGGOaakcqWG3bWDcqWGHbqycqGGPaqkaeaacqWFobGtdaWgaaWcbaGae4xmaedabeaakiabcIcaOiabdEha3jabdggaHjabcMcaPaaacqGHxdaTcqWGqbaucqGGOaakcqWFobGtcqGGPaqkcaWLjaGaaCzcamaabmaabaGaeGymaeJaeGioaGdacaGLOaGaayzkaaaaaa@5324@

If we denote by

*P *= *μ *(*w*_1 _... *w*_*m*_) × *π *(*w*_1 _... *w*_*m*_, *w*_*m*+1_) × ... × *π *(*w*_*h*-*m *_... *w*_*h*-1_, *w*_*h*_)     (19)

the *true *probability for *W *to occur at a given position in the sequence *X *then we get, using (7) in (13), that

P(E)=p×(1−1n−m+1)h−m≃p     (20)
 MathType@MTEF@5@5@+=feaafiart1ev1aaatCvAUfKttLearuWrP9MDH5MBPbIqV92AaeXatLxBI9gBaebbnrfifHhDYfgasaacH8akY=wiFfYdH8Gipec8Eeeu0xXdbba9frFj0=OqFfea0dXdd9vqai=hGuQ8kuc9pgc9s8qqaq=dirpe0xb9q8qiLsFr0=vr0=vr0dc8meaabaqaciaacaGaaeqabaqabeGadaaakeaacqWGqbaucqGGOaakieqacqWFfbqrcqGGPaqkcqGH9aqpcqWGWbaCcqGHxdaTdaqadaqaaiabigdaXiabgkHiTmaalaaabaGaeGymaedabaGaemOBa4MaeyOeI0IaemyBa0Maey4kaSIaeGymaedaaaGaayjkaiaawMcaamaaCaaaleqabaGaemiAaGMaeyOeI0IaemyBa0gaaOGaeS4qISJaemiCaaNaaCzcaiaaxMaadaqadaqaaiabikdaYiabicdaWaGaayjkaiaawMcaaaaa@4A3A@

for *n *large enough. We hence get

∇F+(E)≃pNobs(1−p)ℓh−Nobsβ(Nobs,ℓh−Nobs+1)×G     (21)
 MathType@MTEF@5@5@+=feaafiart1ev1aaatCvAUfKttLearuWrP9MDH5MBPbIqV92AaeXatLxBI9gBaebbnrfifHhDYfgasaacH8akY=wiFfYdH8Gipec8Eeeu0xXdbba9frFj0=OqFfea0dXdd9vqai=hGuQ8kuc9pgc9s8qqaq=dirpe0xb9q8qiLsFr0=vr0=vr0dc8meaabaqaciaacaGaaeqabaqabeGadaaakeaacqGHhis0cqWGgbGrdaahaaWcbeqaaiabgUcaRaaakiabcIcaOGqabiab=veafjabcMcaPiabloKi7maalaaabaGaemiCaa3aaWbaaSqabeaacqWGobGtdaWgaaadbaGaee4Ba8MaeeOyaiMaee4CamhabeaaaaGccqGGOaakcqaIXaqmcqGHsislcqWGWbaCcqGGPaqkdaahaaWcbeqaaiabloriSnaaBaaameaacqWGObaAaeqaaSGaeyOeI0IaemOta40aaSbaaWqaaiabb+gaVjabbkgaIjabbohaZbqabaaaaaGcbaacciGae4NSdiMaeiikaGIaemOta40aaSbaaSqaaiabb+gaVjabbkgaIjabbohaZbqabaGccqGGSaalcqWItecBdaWgaaWcbaGaemiAaGgabeaakiabgkHiTiabd6eaonaaBaaaleaacqqGVbWBcqqGIbGycqqGZbWCaeqaaOGaey4kaSIaeGymaeJaeiykaKcaaiabgEna0kab=DeahjaaxMaacaWLjaWaaeWaaeaacqaIYaGmcqaIXaqmaiaawIcacaGLPaaaaaa@6649@

where ^*t*^**G **= [^*t*^**G**_0 _^*t*^**G**_1_] is defined by

G0(w)=−A0(w)E0(w)andG1(wa)=−A1(wa)E1(wa)     (22)
 MathType@MTEF@5@5@+=feaafiart1ev1aaatCvAUfKttLearuWrP9MDH5MBPbIqV92AaeXatLxBI9gBaebbnrfifHhDYfgasaacH8akY=wiFfYdH8Gipec8Eeeu0xXdbba9frFj0=OqFfea0dXdd9vqai=hGuQ8kuc9pgc9s8qqaq=dirpe0xb9q8qiLsFr0=vr0=vr0dc8meaabaqaciaacaGaaeqabaqabeGadaaakeaafaqaaeqadaaabaacbeGae83raC0aaSbaaSqaaiabicdaWaqabaGccqGGOaakcqWG3bWDcqGGPaqkcqGH9aqpcqGHsisldaWcaaqaaiabdgeabnaaBaaaleaacqaIWaamaeqaaOGaeiikaGIaem4DaCNaeiykaKcabaGae8xrau0aaSbaaSqaaiabicdaWaqabaGccqGGOaakcqWG3bWDcqGGPaqkaaaabaGaeeyyaeMaeeOBa4MaeeizaqgabaGae83raC0aaSbaaSqaaiabigdaXaqabaGccqGGOaakcqWG3bWDcqWGHbqycqGGPaqkcqGH9aqpcqGHsisldaWcaaqaaiabdgeabnaaBaaaleaacqaIXaqmaeqaaOGaeiikaGIaem4DaCNaemyyaeMaeiykaKcabaGae8xrau0aaSbaaSqaaiabigdaXaqabaGccqGGOaakcqWG3bWDcqWGHbqycqGGPaqkaaaaaiaaxMaacaWLjaWaaeWaaeaacqaIYaGmcqaIYaGmaiaawIcacaGLPaaaaaa@5D85@

Using equation (12) we finally get

σ≃Q+ tG×C×G     (23)
 MathType@MTEF@5@5@+=feaafiart1ev1aaatCvAUfKttLearuWrP9MDH5MBPbIqV92AaeXatLxBI9gBaebbnrfifHhDYfgasaacH8akY=wiFfYdH8Gipec8Eeeu0xXdbba9frFj0=OqFfea0dXdd9vqai=hGuQ8kuc9pgc9s8qqaq=dirpe0xb9q8qiLsFr0=vr0=vr0dc8meaabaqaciaacaGaaeqabaqabeGadaaakeaaiiGacqWFdpWCcqWIdjYocqWGrbqudaahaaWcbeqaaiabgUcaRaaakmaakaaabaGaeeiiaaYaaWbaaSqabeaacqWG0baDaaacbeGccqGFhbWrcqGHxdaTcqGFdbWqcqGHxdaTcqGFhbWraSqabaGccaWLjaGaaCzcamaabmaabaGaeGOmaiJaeG4mamdacaGLOaGaayzkaaaaaa@4098@

where

Q+=pNobs(1−p)ℓh−Nobsln⁡(10)β(p,Nobs,ℓh−Nobs+1)     (24)
 MathType@MTEF@5@5@+=feaafiart1ev1aaatCvAUfKttLearuWrP9MDH5MBPbIqV92AaeXatLxBI9gBaebbnrfifHhDYfgasaacH8akY=wiFfYdH8Gipec8Eeeu0xXdbba9frFj0=OqFfea0dXdd9vqai=hGuQ8kuc9pgc9s8qqaq=dirpe0xb9q8qiLsFr0=vr0=vr0dc8meaabaqaciaacaGaaeqabaqabeGadaaakeaacqWGrbqudaahaaWcbeqaaiabgUcaRaaakiabg2da9maalaaabaGaemiCaa3aaWbaaSqabeaacqWGobGtdaWgaaadbaGaee4Ba8MaeeOyaiMaee4CamhabeaaaaGccqGGOaakcqaIXaqmcqGHsislcqWGWbaCcqGGPaqkdaahaaWcbeqaaiabloriSnaaBaaameaacqWGObaAaeqaaSGaeyOeI0IaemOta40aaSbaaWqaaiabb+gaVjabbkgaIjabbohaZbqabaaaaaGcbaGagiiBaWMaeiOBa4MaeiikaGIaeGymaeJaeGimaaJaeiykaKccciGae8NSdiMaeiikaGIaemiCaaNaeiilaWIaemOta40aaSbaaSqaaiabb+gaVjabbkgaIjabbohaZbqabaGccqGGSaalcqWItecBdaWgaaWcbaGaemiAaGgabeaakiabgkHiTiabd6eaonaaBaaaleaacqqGVbWBcqqGIbGycqqGZbWCaeqaaOGaey4kaSIaeGymaeJaeiykaKcaaiaaxMaacaWLjaWaaeWaaeaacqaIYaGmcqaI0aanaiaawIcacaGLPaaaaaa@675F@

and then, a computation of *σ *is possible by plug-in. Without considering the computation of **E **and **C**, the complexity of this approach is *O*(*h*) (where *h *is the size of the pattern).

When a degenerate pattern (finite set of words) is considered instead of a single word, it is easy to adapt this method by summing the contribution *p *of each word belonging to the pattern. This point is left to the reader.

### Under-represented pattern

In the case of an under-represented pattern we have

SN=log⁡10F−(N)withF−(N)≜ℙμN,πN(N≤Nobs).     (25)
 MathType@MTEF@5@5@+=feaafiart1ev1aaatCvAUfKttLearuWrP9MDH5MBPbIqV92AaeXatLxBI9gBaebbnrfifHhDYfgasaacH8akY=wiFfYdH8Gipec8Eeeu0xXdbba9frFj0=OqFfea0dXdd9vqai=hGuQ8kuc9pgc9s8qqaq=dirpe0xb9q8qiLsFr0=vr0=vr0dc8meaabaqaciaacaGaaeqabaqabeGadaaakeaafaqaaeqadaaabaGaem4uam1aaSbaaSqaaGqabiab=5eaobqabaGccqGH9aqpcyGGSbaBcqGGVbWBcqGGNbWzdaWgaaWcbaGaeGymaeJaeGimaadabeaakiabdAeagnaaCaaaleqabaGaeyOeI0caaOGaeiikaGIae8Nta4KaeiykaKcabaGaee4DaCNaeeyAaKMaeeiDaqNaeeiAaGgabaGaemOray0aaWbaaSqabeaacqGHsislaaGccqGGOaakcqWFobGtcqGGPaqkcqWICjcqtuuDJXwAK1uy0HMmaeHbfv3ySLgzG0uy0HgiuD3BaGabaiab+LriqnaaBaaaleaaiiGacqqF8oqBdaWgaaadbaGae8Nta4eabeaaliabcYcaSiab9b8aWnaaBaaameaacqWFobGtaeqaaaWcbeaakiabcIcaOiabd6eaojabgsMiJkabd6eaonaaBaaaleaacqqGVbWBcqqGIbGycqqGZbWCaeqaaOGaeiykaKIaeiOla4IaaCzcaiaaxMaadaqadaqaaiabikdaYiabiwda1aGaayjkaiaawMcaaaaaaaa@6905@

Using a binomial approximation we get

F−(N)≃ℙ(ℬ(ℓh,P(N))≤Nobs)=β−(P(N),Nobs+1,ℓh−Nobs)β(Nobs+1,ℓh−Nobs)     (26)
 MathType@MTEF@5@5@+=feaafiart1ev1aaatCvAUfKttLearuWrP9MDH5MBPbIqV92AaeXatLxBI9gBamrtHrhAL1wy0L2yHvtyaeHbnfgDOvwBHrxAJfwnaebbnrfifHhDYfgasaacH8akY=wiFfYdH8Gipec8Eeeu0xXdbba9frFj0=OqFfea0dXdd9vqai=hGuQ8kuc9pgc9s8qqaq=dirpe0xb9q8qiLsFr0=vr0=vr0dc8meaabaqaciaacaGaaeqabaWaaeGaeaaakeaacqWGgbGrdaahaaWcbeqaaiabgkHiTaaakiabcIcaOGqabiab=5eaojabcMcaPiabloKi7mrr1ngBPrwtHrhAYaqehuuDJXwAKbstHrhAGq1DVbacfaGae4xgHaLaeiikaGccdaGae0hlHiKaeiikaGIaeS4eHW2aaSbaaSqaaiabdIgaObqabaGccqGGSaalcqWGqbaucqGGOaakcqWFobGtcqGGPaqkcqGGPaqkcqGHKjYOcqWGobGtdaWgaaWcbaacbaGaeW3Ba8MaeWNyaiMaeW3CamhabeaakiabcMcaPiabg2da9maalaaabaacciGaeSNSdi2aaWbaaSqabeaacqWEsislaaGccqGGOaakcqWGqbaucqGGOaakcqWFobGtcqGGPaqkcqGGSaalcqWGobGtdaWgaaWcbaGaeW3Ba8MaeWNyaiMaeW3CamhabeaakiabgUcaRiabigdaXiabcYcaSiabloriSnaaBaaaleaacqWGObaAaeqaaOGaeyOeI0IaemOta40aaSbaaSqaaiab89gaVjab8jgaIjab8nhaZbqabaGccqGGPaqkaeaacqWEYoGycqGGOaakcqWGobGtdaWgaaWcbaGaeW3Ba8MaeWNyaiMaeW3CamhabeaakiabgUcaRiabigdaXiabcYcaSiabloriSnaaBaaaleaacqWGObaAaeqaaOGaeyOeI0IaemOta40aaSbaaSqaaiab89gaVjab8jgaIjab8nhaZbqabaGccqGGPaqkaaGaaCzcaiaaxMaacqGGOaakcqaIYaGmcqaI2aGncqGGPaqkaaa@8F8B@

and, by the same method than in the over-represented case we finally have

σ≃Q− tG×C×G     (27)
 MathType@MTEF@5@5@+=feaafiart1ev1aaatCvAUfKttLearuWrP9MDH5MBPbIqV92AaeXatLxBI9gBaebbnrfifHhDYfgasaacH8akY=wiFfYdH8Gipec8Eeeu0xXdbba9frFj0=OqFfea0dXdd9vqai=hGuQ8kuc9pgc9s8qqaq=dirpe0xb9q8qiLsFr0=vr0=vr0dc8meaabaqaciaacaGaaeqabaqabeGadaaakeaaiiGacqWFdpWCcqWIdjYocqWGrbqudaahaaWcbeqaaiabgkHiTaaakmaakaaabaGaeeiiaaYaaWbaaSqabeaaieGacqGF0baDaaacbeGccqqFhbWrcqGHxdaTcqqFdbWqcqGHxdaTcqqFhbWraSqabaGccaWLjaGaaCzcamaabmaabaGaeGOmaiJaeG4naCdacaGLOaGaayzkaaaaaa@40AE@

where

Q−=pNobs+1(1−p)ℓh−Nobs−1ln⁡(10)β−(p,Nobs+1,ℓh−Nobs)     (28)
 MathType@MTEF@5@5@+=feaafiart1ev1aaatCvAUfKttLearuWrP9MDH5MBPbIqV92AaeXatLxBI9gBaebbnrfifHhDYfgasaacH8akY=wiFfYdH8Gipec8Eeeu0xXdbba9frFj0=OqFfea0dXdd9vqai=hGuQ8kuc9pgc9s8qqaq=dirpe0xb9q8qiLsFr0=vr0=vr0dc8meaabaqaciaacaGaaeqabaqabeGadaaakeaacqWGrbqudaahaaWcbeqaaiabgkHiTaaakiabg2da9maalaaabaGaemiCaa3aaWbaaSqabeaacqWGobGtdaWgaaadbaGaee4Ba8MaeeOyaiMaee4CamhabeaaliabgUcaRiabigdaXaaakiabcIcaOiabigdaXiabgkHiTiabdchaWjabcMcaPmaaCaaaleqabaGaeS4eHW2aaSbaaWqaaiabdIgaObqabaWccqGHsislcqWGobGtdaWgaaadbaGaee4Ba8MaeeOyaiMaee4CamhabeaaliabgkHiTiabigdaXaaaaOqaaiGbcYgaSjabc6gaUjabcIcaOiabigdaXiabicdaWiabcMcaPGGaciab=j7aInaaCaaaleqabaGaeyOeI0caaOGaeiikaGIaemiCaaNaeiilaWIaemOta40aaSbaaSqaaiabb+gaVjabbkgaIjabbohaZbqabaGccqGHRaWkcqaIXaqmcqGGSaalcqWItecBdaWgaaWcbaGaemiAaGgabeaakiabgkHiTiabd6eaonaaBaaaleaacqqGVbWBcqqGIbGycqqGZbWCaeqaaOGaeiykaKcaaiaaxMaacaWLjaWaaeWaaeaacqaIYaGmcqaI4aaoaiaawIcacaGLPaaaaaa@6C5B@

### Two distinct patterns

We consider now two patterns *V *and *W *instead of one and want to study the joint distribution of *S*_**N **_(*V*) and *S*_**N **_(*W*) their corresponding pattern statistics.

With a similar argument as in section "delta method", it is easy to show that

ℒ([SN(V)SN(W)])≃N([S(V)S(W)],[σV2σV,WσV,WσW2])     (29)
 MathType@MTEF@5@5@+=feaafiart1ev1aaatCvAUfKttLearuWrP9MDH5MBPbIqV92AaeXatLxBI9gBamrtHrhAL1wy0L2yHvtyaeHbnfgDOvwBHrxAJfwnaebbnrfifHhDYfgasaacH8akY=wiFfYdH8Gipec8Eeeu0xXdbba9frFj0=OqFfea0dXdd9vqai=hGuQ8kuc9pgc9s8qqaq=dirpe0xb9q8qiLsFr0=vr0=vr0dc8meaabaqaciaacaGaaeqabaWaaeGaeaaakeaaimaacqWFsectdaqadaqaamaadmaabaqbaeqabiqaaaqaaiabdofatnaaBaaaleaaieqacqGFobGtaeqaaOGaeiikaGIaemOvayLaeiykaKcabaGaem4uam1aaSbaaSqaaiab+5eaobqabaGccqGGOaakcqWGxbWvcqGGPaqkaaaacaGLBbGaayzxaaaacaGLOaGaayzkaaGaeS4qISJae8xdX70aaeWaaeaadaWadaqaauaabeqaceaaaeaacqWGtbWucqGGOaakcqWGwbGvcqGGPaqkaeaacqWGtbWucqGGOaakcqWGxbWvcqGGPaqkaaaacaGLBbGaayzxaaGaeiilaWYaamWaaeaafaqabeGacaaabaacciGae03Wdm3aa0baaSqaaiabdAfawbqaaiabikdaYaaaaOqaaiab9n8aZnaaBaaaleaacqWGwbGvcqGGSaalcqWGxbWvaeqaaaGcbaGae03Wdm3aaSbaaSqaaiabdAfawjabcYcaSiabdEfaxbqabaaakeaacqqFdpWCdaqhaaWcbaGaem4vaCfabaGaeGOmaidaaaaaaOGaay5waiaaw2faaaGaayjkaiaawMcaaiaaxMaacaWLjaGaeiikaGIaeGOmaiJaeGyoaKJaeiykaKcaaa@6F1A@

where *σ*_*V *_(resp. *σ*_*W*_) is the standard deviation *σ *for the pattern *V *(resp. *W*) and where

σV,W= t∇FVε(E)×C×∇FWη(E)ln⁡(10)FVε(E)×ln⁡(10)FWη(E)     (30)
 MathType@MTEF@5@5@+=feaafiart1ev1aaatCvAUfKttLearuWrP9MDH5MBPbIqV92AaeXatLxBI9gBaebbnrfifHhDYfgasaacH8akY=wiFfYdH8Gipec8Eeeu0xXdbba9frFj0=OqFfea0dXdd9vqai=hGuQ8kuc9pgc9s8qqaq=dirpe0xb9q8qiLsFr0=vr0=vr0dc8meaabaqaciaacaGaaeqabaqabeGadaaakeaaiiGacqWFdpWCdaWgaaWcbaGaemOvayLaeiilaWIaem4vaCfabeaakiabg2da9maalaaabaGaeeiiaaYaaWbaaSqabeaaieGacqGF0baDaaGccqGHhis0cqWGgbGrdaqhaaWcbaGaemOvayfabaGae8xTdugaaOGaeiikaGccbeGae0xrauKaeiykaKIaey41aqRae03qamKaey41aqRaey4bIeTaemOray0aa0baaSqaaiabdEfaxbqaaiab=D7aObaakiabcIcaOiab9veafjabcMcaPaqaaiGbcYgaSjabc6gaUjabcIcaOiabigdaXiabicdaWiabcMcaPiabdAeagnaaDaaaleaacqWGwbGvaeaacqWF1oqzaaGccqGGOaakcqqFfbqrcqGGPaqkcqGHxdaTcyGGSbaBcqGGUbGBcqGGOaakcqaIXaqmcqaIWaamcqGGPaqkcqWGgbGrdaqhaaWcbaGaem4vaCfabaGae83TdGgaaOGaeiikaGIae0xrauKaeiykaKcaaiaaxMaacaWLjaWaaeWaaeaacqaIZaWmcqaIWaamaiaawIcacaGLPaaaaaa@6CD8@

where

ε (resp. η)={+if pattern V (resp. W) is over-represented−if pattern V (resp. W) is unter-represented.     (31)
 MathType@MTEF@5@5@+=feaafiart1ev1aaatCvAUfKttLearuWrP9MDH5MBPbIqV92AaeXatLxBI9gBaebbnrfifHhDYfgasaacH8akY=wiFfYdH8Gipec8Eeeu0xXdbba9frFj0=OqFfea0dXdd9vqai=hGuQ8kuc9pgc9s8qqaq=dirpe0xb9q8qiLsFr0=vr0=vr0dc8meaabaqaciaacaGaaeqabaqabeGadaaakeaaiiGacqWF1oqzcqqGGaaicqGGOaakcqqGYbGCcqqGLbqzcqqGZbWCcqqGWbaCcqGGUaGlcqqGGaaicqWF3oaAcqGGPaqkcqGH9aqpdaGabeqaauaabaqaciaaaeaacqGHRaWkaeaacqqGPbqAcqqGMbGzcqqGGaaicqqGWbaCcqqGHbqycqqG0baDcqqG0baDcqqGLbqzcqqGYbGCcqqGUbGBcqqGGaaicqWGwbGvcqqGGaaicqGGOaakcqqGYbGCcqqGLbqzcqqGZbWCcqqGWbaCcqqGUaGlcqqGGaaicqWGxbWvcqGGPaqkcqqGGaaicqqGPbqAcqqGZbWCcqqGGaaicqqGVbWBcqqG2bGDcqqGLbqzcqqGYbGCcqqGTaqlcqqGYbGCcqqGLbqzcqqGWbaCcqqGYbGCcqqGLbqzcqqGZbWCcqqGLbqzcqqGUbGBcqqG0baDcqqGLbqzcqqGKbazaeaacqGHsislaeaacqqGPbqAcqqGMbGzcqqGGaaicqqGWbaCcqqGHbqycqqG0baDcqqG0baDcqqGLbqzcqqGYbGCcqqGUbGBcqqGGaaicqWGwbGvcqqGGaaicqGGOaakcqqGYbGCcqqGLbqzcqqGZbWCcqqGWbaCcqqGUaGlcqqGGaaicqWGxbWvcqGGPaqkcqqGGaaicqqGPbqAcqqGZbWCcqqGGaaicqqG1bqDcqqGUbGBcqqG0baDcqqGLbqzcqqGYbGCcqqGTaqlcqqGYbGCcqqGLbqzcqqGWbaCcqqGYbGCcqqGLbqzcqqGZbWCcqqGLbqzcqqGUbGBcqqG0baDcqqGLbqzcqqGKbazaaaacaGL7baacqGGUaGlcaWLjaGaaCzcamaabmaabaGaeG4mamJaeGymaedacaGLOaGaayzkaaaaaa@AC70@

And after using results of sections "single pattern" and "under-represented pattern" we finally get

σV,W=(QVεQWη)×( t∇GV×C×∇GW)     (32)
 MathType@MTEF@5@5@+=feaafiart1ev1aaatCvAUfKttLearuWrP9MDH5MBPbIqV92AaeXatLxBI9gBaebbnrfifHhDYfgasaacH8akY=wiFfYdH8Gipec8Eeeu0xXdbba9frFj0=OqFfea0dXdd9vqai=hGuQ8kuc9pgc9s8qqaq=dirpe0xb9q8qiLsFr0=vr0=vr0dc8meaabaqaciaacaGaaeqabaqabeGadaaakeaaiiGacqWFdpWCdaWgaaWcbaGaemOvayLaeiilaWIaem4vaCfabeaakiabg2da9maabmaabaGaemyuae1aa0baaSqaaiabdAfawbqaaiab=v7aLbaakiabdgfarnaaDaaaleaacqWGxbWvaeaacqWF3oaAaaaakiaawIcacaGLPaaacqGHxdaTdaqadaqaaiabbccaGmaaCaaaleqabaGaemiDaqhaaOGaey4bIencbeGae43raC0aaSbaaSqaaiabdAfawbqabaGccqGHxdaTcqGFdbWqcqGHxdaTcqGHhis0cqGFhbWrdaWgaaWcbaGaem4vaCfabeaaaOGaayjkaiaawMcaaiaaxMaacaWLjaWaaeWaaeaacqaIZaWmcqaIYaGmaiaawIcacaGLPaaaaaa@550C@

where QVε
 MathType@MTEF@5@5@+=feaafiart1ev1aaatCvAUfKttLearuWrP9MDH5MBPbIqV92AaeXatLxBI9gBaebbnrfifHhDYfgasaacH8akY=wiFfYdH8Gipec8Eeeu0xXdbba9frFj0=OqFfea0dXdd9vqai=hGuQ8kuc9pgc9s8qqaq=dirpe0xb9q8qiLsFr0=vr0=vr0dc8meaabaqaciaacaGaaeqabaqabeGadaaakeaacqWGrbqudaqhaaWcbaGaemOvayfabaacciGae8xTdugaaaaa@30E7@ (resp. *W*) and **G**_*V *_(resp. *W*) are the constant *Q *(*Q*^+ ^and *Q*^-^) and the vector **G **for the pattern *V *(resp. *W*).

### Simulations

It is also possible to study the empirical distribution of a *S*_**N **_(for one or more patterns) through simulations.

In order to do so, we first draw *M *independent sequences *Y*^*j *^= Y1j
 MathType@MTEF@5@5@+=feaafiart1ev1aaatCvAUfKttLearuWrP9MDH5MBPbIqV92AaeXatLxBI9gBaebbnrfifHhDYfgasaacH8akY=wiFfYdH8Gipec8Eeeu0xXdbba9frFj0=OqFfea0dXdd9vqai=hGuQ8kuc9pgc9s8qqaq=dirpe0xb9q8qiLsFr0=vr0=vr0dc8meaabaqaciaacaGaaeqabaqabeGadaaakeaacqWGzbqwdaqhaaWcbaGaeGymaedabaGaemOAaOgaaaaa@3061@ ... Ynj
 MathType@MTEF@5@5@+=feaafiart1ev1aaatCvAUfKttLearuWrP9MDH5MBPbIqV92AaeXatLxBI9gBaebbnrfifHhDYfgasaacH8akY=wiFfYdH8Gipec8Eeeu0xXdbba9frFj0=OqFfea0dXdd9vqai=hGuQ8kuc9pgc9s8qqaq=dirpe0xb9q8qiLsFr0=vr0=vr0dc8meaabaqaciaacaGaaeqabaqabeGadaaakeaacqWGzbqwdaqhaaWcbaGaemOBa4gabaGaemOAaOgaaaaa@30D6@ using an order *m *stationary Markov model of parameters *π*. Complexity of this step is *O*(*M *× *n*).

For each *j *we get the frequencies **N**^*j *^= (N0j
 MathType@MTEF@5@5@+=feaafiart1ev1aaatCvAUfKttLearuWrP9MDH5MBPbIqV92AaeXatLxBI9gBaebbnrfifHhDYfgasaacH8akY=wiFfYdH8Gipec8Eeeu0xXdbba9frFj0=OqFfea0dXdd9vqai=hGuQ8kuc9pgc9s8qqaq=dirpe0xb9q8qiLsFr0=vr0=vr0dc8meaabaqaciaacaGaaeqabaqabeGadaaakeaaieqacqWFobGtdaqhaaWcbaGaeGimaadabaGaemOAaOgaaaaa@304F@,N1j
 MathType@MTEF@5@5@+=feaafiart1ev1aaatCvAUfKttLearuWrP9MDH5MBPbIqV92AaeXatLxBI9gBaebbnrfifHhDYfgasaacH8akY=wiFfYdH8Gipec8Eeeu0xXdbba9frFj0=OqFfea0dXdd9vqai=hGuQ8kuc9pgc9s8qqaq=dirpe0xb9q8qiLsFr0=vr0=vr0dc8meaabaqaciaacaGaaeqabaqabeGadaaakeaaieqacqWFobGtdaqhaaWcbaGaeGymaedabaGaemOAaOgaaaaa@3051@) (with complexity *O*(*n*) for each sequence) of the words of size *m *and *m *+ 1 in the sequence *Y*^*j *^and use it to compute *S*^*j *^= SNj
 MathType@MTEF@5@5@+=feaafiart1ev1aaatCvAUfKttLearuWrP9MDH5MBPbIqV92AaeXatLxBI9gBaebbnrfifHhDYfgasaacH8akY=wiFfYdH8Gipec8Eeeu0xXdbba9frFj0=OqFfea0dXdd9vqai=hGuQ8kuc9pgc9s8qqaq=dirpe0xb9q8qiLsFr0=vr0=vr0dc8meaabaqaciaacaGaaeqabaqabeGadaaakeaacqWGtbWudaWgaaWcbaacbeGae8Nta40aaWbaaWqabeaacqWGQbGAaaaaleqaaaaa@30C8@ (exact value or approximation). Complexity here depends on the statistical method used to compute *S*^*j *^(*e.g*. *O*(*h*) using a binomial approximation).

We now have a *M *– sample *S*^1^, ..., *S*^*M *^of *S*_**N **_from which we can easily estimate *σ *and thus, valid or invalid the approximation through the delta-method.

When used with large value of *n *(*e.g*. several millions or more), the complexity of this approach is slowed by the drawn of the sequences *Y*_*j*_. It is therefore possible to improve the method by simulating directly the frequencies **N **through (5). As this approximation has a very small impact on the distribution of *S*_**N **_(data not shown) it may dramatically speed-up the computations when considering large *n *or *M*. It is nevertheless important to point out that drawing a Gaussian vector size *L *requires to precompute the Choleski decomposition of its covariance matrix which could be a limiting factor when considering large *L*.

## Results and discussion

### Validation

#### Simple case

Let us start with a simple case: a binary alphabet A
 MathType@MTEF@5@5@+=feaafiart1ev1aaatCvAUfKttLearuWrP9MDH5MBPbIqV92AaeXatLxBI9gBamrtHrhAL1wy0L2yHvtyaeHbnfgDOvwBHrxAJfwnaebbnrfifHhDYfgasaacH8akY=wiFfYdH8Gipec8Eeeu0xXdbba9frFj0=OqFfea0dXdd9vqai=hGuQ8kuc9pgc9s8qqaq=dirpe0xb9q8qiLsFr0=vr0=vr0dc8meaabaqaciaacaGaaeqabaWaaeGaeaaakeaaimaacqWFaeFqaaa@3821@ = {a, b} (*k *= 2) with an order *m *= 1 Markov model

π=(0.30.70.60.4)     (33)
 MathType@MTEF@5@5@+=feaafiart1ev1aaatCvAUfKttLearuWrP9MDH5MBPbIqV92AaeXatLxBI9gBaebbnrfifHhDYfgasaacH8akY=wiFfYdH8Gipec8Eeeu0xXdbba9frFj0=OqFfea0dXdd9vqai=hGuQ8kuc9pgc9s8qqaq=dirpe0xb9q8qiLsFr0=vr0=vr0dc8meaabaqaciaacaGaaeqabaqabeGadaaakeaaiiGacqWFapaCcqGH9aqpdaqadaqaauaabeqaciaaaeaacqaIWaamcqGGUaGlcqaIZaWmaeaacqaIWaamcqGGUaGlcqaI3aWnaeaacqaIWaamcqGGUaGlcqaI2aGnaeaacqaIWaamcqGGUaGlcqaI0aanaaaacaGLOaGaayzkaaGaaCzcaiaaxMaadaqadaqaaiabiodaZiabiodaZaGaayjkaiaawMcaaaaa@40EC@

which stationary distribution is *μ *= (6/13,7/13) and we work on a sequence of length *n *= 10 000.

The first thing to do is to compute **E **and **C **(see appendix A for details).

Now, we consider the pattern *W *= ababa occurring *N*_obs _= 1221 times in a sequence of length ℓ = *n *= 10 000. We have

*p *= *μ*(a) Π (a,b)^2 ^Π (b,a)^2 ^= 8.142 × 10^-2 ^    (34)

so E
 MathType@MTEF@5@5@+=feaafiart1ev1aaatCvAUfKttLearuWrP9MDH5MBPbIqV92AaeXatLxBI9gBaebbnrfifHhDYfgasaacH8akY=wiFfYdH8Gipec8Eeeu0xXdbba9frFj0=OqFfea0dXdd9vqai=hGuQ8kuc9pgc9s8qqaq=dirpe0xb9q8qiLsFr0=vr0=vr0dc8meaabaqaciaacaGaaeqabaqabeGadaaakeaatuuDJXwAK1uy0HMmaeHbfv3ySLgzG0uy0HgiuD3BaGabaiab=ri8fbaa@388C@[*N*(ababa)] = (ℓ - 4)*p *= 813.8 ≃ 0.66 × *N*_obs _and hence the pattern is over-represented. Its statistic (using binomial approximation) is

S≃−log⁡10ℙ(ℬ(ℓ−5+1,p)≥Nobs)=43.74285     (35)
 MathType@MTEF@5@5@+=feaafiart1ev1aaatCvAUfKttLearuWrP9MDH5MBPbIqV92AaeXatLxBI9gBamrtHrhAL1wy0L2yHvtyaeHbnfgDOvwBHrxAJfwnaebbnrfifHhDYfgasaacH8akY=wiFfYdH8Gipec8Eeeu0xXdbba9frFj0=OqFfea0dXdd9vqai=hGuQ8kuc9pgc9s8qqaq=dirpe0xb9q8qiLsFr0=vr0=vr0dc8meaabaqaciaacaGaaeqabaWaaeGaeaaakeaacqWGtbWucqWIdjYocqGHsislcyGGSbaBcqGGVbWBcqGGNbWzdaWgaaWcbaGaeGymaeJaeGimaadabeaatuuDJXwAK1uy0HMmaeXbfv3ySLgzG0uy0HgiuD3BaGqbaOGae8xgHaLaeiikaGccdaGae4hlHiKaeiikaGIaeS4eHWMaeyOeI0IaeGynauJaey4kaSIaeGymaeJaeiilaWIaemiCaaNaeiykaKIaeyyzImRaemOta40aaSbaaSqaaGqaaiab99gaVjab9jgaIjab9nhaZbqabaGccqGGPaqkcqGH9aqpcqaI0aancqaIZaWmcqGGUaGlcqaI3aWncqaI0aancqaIYaGmcqaI4aaocqaI1aqncaWLjaGaaCzcaiabcIcaOiabiodaZiabiwda1iabcMcaPaaa@6B0F@

We have

Q+=pNobs−1(1−p)ℓ−4−Nobsln⁡(10)β(p,Nobs,ℓ−3−Nobs)=193.3258     (36)
 MathType@MTEF@5@5@+=feaafiart1ev1aaatCvAUfKttLearuWrP9MDH5MBPbIqV92AaeXatLxBI9gBaebbnrfifHhDYfgasaacH8akY=wiFfYdH8Gipec8Eeeu0xXdbba9frFj0=OqFfea0dXdd9vqai=hGuQ8kuc9pgc9s8qqaq=dirpe0xb9q8qiLsFr0=vr0=vr0dc8meaabaqaciaacaGaaeqabaqabeGadaaakeaacqWGrbqudaahaaWcbeqaaiabgUcaRaaakiabg2da9maalaaabaGaemiCaa3aaWbaaSqabeaacqWGobGtdaWgaaadbaGaee4Ba8MaeeOyaiMaee4CamhabeaaliabgkHiTiabigdaXaaakiabcIcaOiabigdaXiabgkHiTiabdchaWjabcMcaPmaaCaaaleqabaGaeS4eHWMaeyOeI0IaeGinaqJaeyOeI0IaemOta40aaSbaaWqaaiabb+gaVjabbkgaIjabbohaZbqabaaaaaGcbaGagiiBaWMaeiOBa4MaeiikaGIaeGymaeJaeGimaaJaeiykaKccciGae8NSdiMaeiikaGIaemiCaaNaeiilaWIaemOta40aaSbaaSqaaiabb+gaVjabbkgaIjabbohaZbqabaGccqGGSaalcqWItecBcqGHsislcqaIZaWmcqGHsislcqWGobGtdaWgaaWcbaGaee4Ba8MaeeOyaiMaee4CamhabeaakiabcMcaPaaacqGH9aqpcqaIXaqmcqaI5aqocqaIZaWmcqGGUaGlcqaIZaWmcqaIYaGmcqaI1aqncqaI4aaocaWLjaGaaCzcamaabmaabaGaeG4mamJaeGOnaydacaGLOaGaayzkaaaaaa@70C9@

and

 tG0=[−1E0(a)−2E0(b)]=[−2.17×10−5−3.71×10−5]     (37)
 MathType@MTEF@5@5@+=feaafiart1ev1aaatCvAUfKttLearuWrP9MDH5MBPbIqV92AaeXatLxBI9gBaebbnrfifHhDYfgasaacH8akY=wiFfYdH8Gipec8Eeeu0xXdbba9frFj0=OqFfea0dXdd9vqai=hGuQ8kuc9pgc9s8qqaq=dirpe0xb9q8qiLsFr0=vr0=vr0dc8meaabaqaciaacaGaaeqabaqabeGadaaakeaacqqGGaaidaahaaWcbeqaaiabdsha0baaieqakiab=DeahnaaBaaaleaacqaIWaamaeqaaOGaeyypa0ZaamWaaeaafaqabeqacaaabaWaaSaaaeaacqGHsislcqaIXaqmaeaacqWFfbqrdaWgaaWcbaGaeGimaadabeaakiabcIcaOiabbggaHjabcMcaPaaaaeaadaWcaaqaaiabgkHiTiabikdaYaqaaiab=veafnaaBaaaleaacqaIWaamaeqaaOGaeiikaGIaeeOyaiMaeiykaKcaaaaaaiaawUfacaGLDbaacqGH9aqpdaWadaqaauaabeqabiaaaeaacqGHsislcqaIYaGmcqGGUaGlcqaIXaqmcqaI3aWncqGHxdaTcqaIXaqmcqaIWaamdaahaaWcbeqaaiabgkHiTiabiwda1aaaaOqaaiabgkHiTiabiodaZiabc6caUiabiEda3iabigdaXiabgEna0kabigdaXiabicdaWmaaCaaaleqabaGaeyOeI0IaeGynaudaaaaaaOGaay5waiaaw2faaiaaxMaacaWLjaWaaeWaaeaacqaIZaWmcqaI3aWnaiaawIcacaGLPaaaaaa@5FDF@

and

 tG1=[02E1(ab)2E1(ba)0]=[06.19×10−56.19×10−50]     (38)
 MathType@MTEF@5@5@+=feaafiart1ev1aaatCvAUfKttLearuWrP9MDH5MBPbIqV92AaeXatLxBI9gBaebbnrfifHhDYfgasaacH8akY=wiFfYdH8Gipec8Eeeu0xXdbba9frFj0=OqFfea0dXdd9vqai=hGuQ8kuc9pgc9s8qqaq=dirpe0xb9q8qiLsFr0=vr0=vr0dc8meaabaqaciaacaGaaeqabaqabeGadaaakeaacqqGGaaidaahaaWcbeqaaiabdsha0baaieqakiab=DeahnaaBaaaleaacqaIXaqmaeqaaOGaeyypa0ZaamWaaeaafaqabeqaeaaaaeaacqaIWaamaeaadaWcaaqaaiabikdaYaqaaiab=veafnaaBaaaleaacqaIXaqmaeqaaOGaeiikaGIaeeyyaeMaeeOyaiMaeiykaKcaaaqaamaalaaabaGaeGOmaidabaGae8xrau0aaSbaaSqaaiabigdaXaqabaGccqGGOaakcqqGIbGycqqGHbqycqGGPaqkaaaabaGaeGimaadaaaGaay5waiaaw2faaiabg2da9maadmaabaqbaeqabeabaaaabaGaeGimaadabaGaeGOnayJaeiOla4IaeGymaeJaeGyoaKJaey41aqRaeGymaeJaeGimaaZaaWbaaSqabeaacqGHsislcqaI1aqnaaaakeaacqaI2aGncqGGUaGlcqaIXaqmcqaI5aqocqGHxdaTcqaIXaqmcqaIWaamdaahaaWcbeqaaiabgkHiTiabiwda1aaaaOqaaiabicdaWaaaaiaawUfacaGLDbaacaWLjaGaaCzcamaabmaabaGaeG4mamJaeGioaGdacaGLOaGaayzkaaaaaa@629F@

Finally, we get

σ=Q+ tG×C×G=6.1020774     (39)
 MathType@MTEF@5@5@+=feaafiart1ev1aaatCvAUfKttLearuWrP9MDH5MBPbIqV92AaeXatLxBI9gBaebbnrfifHhDYfgasaacH8akY=wiFfYdH8Gipec8Eeeu0xXdbba9frFj0=OqFfea0dXdd9vqai=hGuQ8kuc9pgc9s8qqaq=dirpe0xb9q8qiLsFr0=vr0=vr0dc8meaabaqaciaacaGaaeqabaqabeGadaaakeaaiiGacqWFdpWCcqGH9aqpcqWGrbqudaahaaWcbeqaaiabgUcaRaaakmaakaaabaGaeeiiaaYaaWbaaSqabeaaieGacqGF0baDaaacbeGccqqFhbWrcqGHxdaTcqqFdbWqcqGHxdaTcqqFhbWraSqabaGccqGH9aqpcqaI2aGncqGGUaGlcqaIXaqmcqaIWaamcqaIYaGmcqaIWaamcqaI3aWncqaI3aWncqaI0aancaWLjaGaaCzcamaabmaabaGaeG4mamJaeGyoaKdacaGLOaGaayzkaaaaaa@4A0E@

As our pattern statistics is the decimal logarithm of the p-value, *σ *= 6 means that the ratio of the estimated p-value over the true one could easily range from 10^-12 ^(10^-2 × *σ*^) to 10^12 ^(10^2 × *σ*^) which is huge.

**Figure 1:**
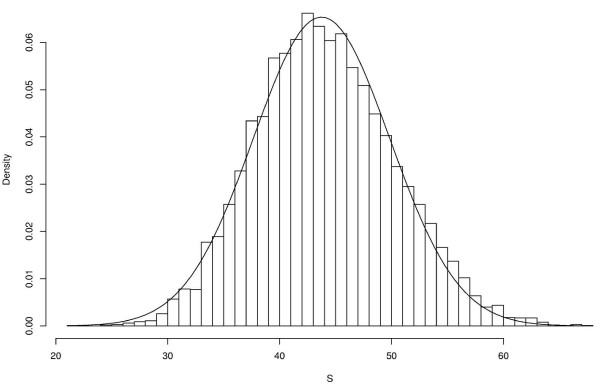
**Empirical and theoretical distributions of **S^
 MathType@MTEF@5@5@+=feaafiart1ev1aaatCvAUfKttLearuWrP9MDH5MBPbIqV92AaeXatLxBI9gBaebbnrfifHhDYfgasaacH8akY=wiFfYdH8Gipec8Eeeu0xXdbba9frFj0=OqFfea0dXdd9vqai=hGuQ8kuc9pgc9s8qqaq=dirpe0xb9q8qiLsFr0=vr0=vr0dc8meaabaqaciaacaGaaeqabaqabeGadaaakeaacuWGtbWugaqcaaaa@2DEB@. A sample of size 10 000 have been used to get the empirical distribution. The solid line represents the density of N
 MathType@MTEF@5@5@+=feaafiart1ev1aaatCvAUfKttLearuWrP9MDH5MBPbIqV92AaeXatLxBI9gBamrtHrhAL1wy0L2yHvtyaeHbnfgDOvwBHrxAJfwnaebbnrfifHhDYfgasaacH8akY=wiFfYdH8Gipec8Eeeu0xXdbba9frFj0=OqFfea0dXdd9vqai=hGuQ8kuc9pgc9s8qqaq=dirpe0xb9q8qiLsFr0=vr0=vr0dc8meaabaqaciaacaGaaeqabaWaaeGaeaaakeaaimaacqWFneVtaaa@383B@(*S*, *σ*^2^). The adjustment test of Kolmogorov-Smirnov give *D *= 0.023 which corresponds to a p-value of *p *= 5.3 × 10^-5^. *N*_obs _= 1221 and *n *= ℓ = 10 000.

We can see on fig. 1 the empirical distribution of *S*_**N **_compared to the theoretical distribution. Even if the two distributions are closely related, an adjustment test (Kolmogorov-Smirnov) shows that they are different.

**Figure 2:**
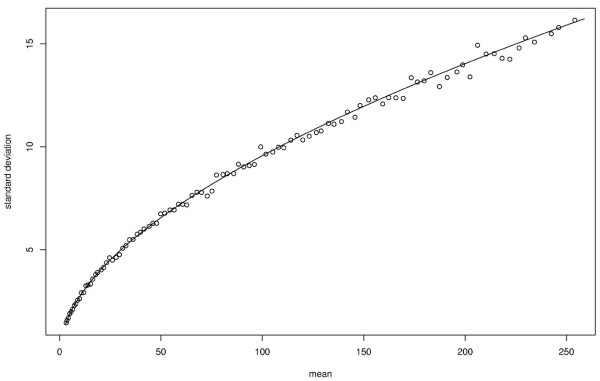
**Comparison of *σ *and **σ^
 MathType@MTEF@5@5@+=feaafiart1ev1aaatCvAUfKttLearuWrP9MDH5MBPbIqV92AaeXatLxBI9gBaebbnrfifHhDYfgasaacH8akY=wiFfYdH8Gipec8Eeeu0xXdbba9frFj0=OqFfea0dXdd9vqai=hGuQ8kuc9pgc9s8qqaq=dirpe0xb9q8qiLsFr0=vr0=vr0dc8meaabaqaciaacaGaaeqabaqabeGadaaakeaaiiGacuWFdpWCgaqcaaaa@2E86@. σ^
 MathType@MTEF@5@5@+=feaafiart1ev1aaatCvAUfKttLearuWrP9MDH5MBPbIqV92AaeXatLxBI9gBaebbnrfifHhDYfgasaacH8akY=wiFfYdH8Gipec8Eeeu0xXdbba9frFj0=OqFfea0dXdd9vqai=hGuQ8kuc9pgc9s8qqaq=dirpe0xb9q8qiLsFr0=vr0=vr0dc8meaabaqaciaacaGaaeqabaqabeGadaaakeaaiiGacuWFdpWCgaqcaaaa@2E86@ is estimated with a sample of size 1 000 and *N*_obs _takes its values from 900 to 1 900. The solid line represents the theoretical values and the circles the empirical ones. The statistic *S *is used on the x-axis. *n *= ℓ = 10 000.

In the fig. 2 we compare *σ *to its estimator σ^
 MathType@MTEF@5@5@+=feaafiart1ev1aaatCvAUfKttLearuWrP9MDH5MBPbIqV92AaeXatLxBI9gBaebbnrfifHhDYfgasaacH8akY=wiFfYdH8Gipec8Eeeu0xXdbba9frFj0=OqFfea0dXdd9vqai=hGuQ8kuc9pgc9s8qqaq=dirpe0xb9q8qiLsFr0=vr0=vr0dc8meaabaqaciaacaGaaeqabaqabeGadaaakeaaiiGacuWFdpWCgaqcaaaa@2E86@ for several values of *N*_obs_. We can see that our theoretical values of *σ *fits very well to the empirical ones.

The equation (39) gives an explicit expression of *σ *as a product of two terms. Once the pattern and the true parameter *π *are fixed, the first term (*Q*) depends only on ℓ and *N*_obs _while the second one only depends on the length *n *of the sequence used for the parameter estimation (see appendix C for an explicit expression of *σ *in the particular case of an order 0 Markov model).

To study the variations of *σ*(*n*) as a function of *n *we therefore need to study **G**(*n*) and **C**(*n*). Using equations (6) and (22) we get that

E(n)=O(n)andG(n)=O(1n)     (40)
 MathType@MTEF@5@5@+=feaafiart1ev1aaatCvAUfKttLearuWrP9MDH5MBPbIqV92AaeXatLxBI9gBaebbnrfifHhDYfgasaacH8akY=wiFfYdH8Gipec8Eeeu0xXdbba9frFj0=OqFfea0dXdd9vqai=hGuQ8kuc9pgc9s8qqaq=dirpe0xb9q8qiLsFr0=vr0=vr0dc8meaabaqaciaacaGaaeqabaqabeGadaaakeaafaqabeqadaaabaacbeGae8xrauKaeiikaGIaemOBa4MaeiykaKIaeyypa0Jaem4ta8KaeiikaGIaemOBa4MaeiykaKcabaGaeeyyaeMaeeOBa4MaeeizaqgabaGae83raCKaeiikaGIaemOBa4MaeiykaKIaeyypa0Jaem4ta80aaeWaaeaadaWcaaqaaiabigdaXaqaaiabd6gaUbaaaiaawIcacaGLPaaaaaGaaCzcaiaaxMaadaqadaqaaiabisda0iabicdaWaGaayjkaiaawMcaaaaa@4920@

Using equations (57) and (58) in appendix A we also get that **C **= **M **+ **O **+ ^*t *^**EE **with

**M**(*n*) = *O*(*n*^2^) and **O**(*n*) = *O*(*n*)     (41)

so finally

σ(n)≃σ˜(n)=Q+×A+Bn     (42)
 MathType@MTEF@5@5@+=feaafiart1ev1aaatCvAUfKttLearuWrP9MDH5MBPbIqV92AaeXatLxBI9gBaebbnrfifHhDYfgasaacH8akY=wiFfYdH8Gipec8Eeeu0xXdbba9frFj0=OqFfea0dXdd9vqai=hGuQ8kuc9pgc9s8qqaq=dirpe0xb9q8qiLsFr0=vr0=vr0dc8meaabaqaciaacaGaaeqabaqabeGadaaakeaaiiGacqWFdpWCcqGGOaakcqWGUbGBcqGGPaqkcqWIdjYocuWFdpWCgaacaiabcIcaOiabd6gaUjabcMcaPiabg2da9iabdgfarnaaCaaaleqabaGaey4kaScaaOGaey41aq7aaOaaaeaacqWGbbqqcqGHRaWkdaWcaaqaaiabdkeacbqaaiabd6gaUbaaaSqabaGccaWLjaGaaCzcamaabmaabaGaeGinaqJaeGOmaidacaGLOaGaayzkaaaaaa@464C@

for large *n*, with

A=lim⁡n→+∞ tG(C−O)G     (43)
 MathType@MTEF@5@5@+=feaafiart1ev1aaatCvAUfKttLearuWrP9MDH5MBPbIqV92AaeXatLxBI9gBaebbnrfifHhDYfgasaacH8akY=wiFfYdH8Gipec8Eeeu0xXdbba9frFj0=OqFfea0dXdd9vqai=hGuQ8kuc9pgc9s8qqaq=dirpe0xb9q8qiLsFr0=vr0=vr0dc8meaabaqaciaacaGaaeqabaqabeGadaaakeaacqWGbbqqcqGH9aqpdaWfqaqaaiGbcYgaSjabcMgaPjabc2gaTbWcbaGaemOBa4MaeyOKH4Qaey4kaSIaeyOhIukabeaakiabbccaGmaaCaaaleqabaGaemiDaqhaaGqabOGae83raCKaeiikaGIae83qamKaeyOeI0Iae83ta8KaeiykaKIae83raCKaaCzcaiaaxMaadaqadaqaaiabisda0iabiodaZaGaayjkaiaawMcaaaaa@46E6@

and

B=lim⁡n→+∞n× tGOG     (44)
 MathType@MTEF@5@5@+=feaafiart1ev1aaatCvAUfKttLearuWrP9MDH5MBPbIqV92AaeXatLxBI9gBaebbnrfifHhDYfgasaacH8akY=wiFfYdH8Gipec8Eeeu0xXdbba9frFj0=OqFfea0dXdd9vqai=hGuQ8kuc9pgc9s8qqaq=dirpe0xb9q8qiLsFr0=vr0=vr0dc8meaabaqaciaacaGaaeqabaqabeGadaaakeaacqWGcbGqcqGH9aqpdaWfqaqaaiGbcYgaSjabcMgaPjabc2gaTbWcbaGaemOBa4MaeyOKH4Qaey4kaSIaeyOhIukabeaakiabd6gaUjabgEna0kabbccaGmaaCaaaleqabaGaemiDaqhaaGqabOGae83raCKae83ta8Kae83raCKaaCzcaiaaxMaadaqadaqaaiabisda0iabisda0aGaayjkaiaawMcaaaaa@46BC@

**Figure 3:**
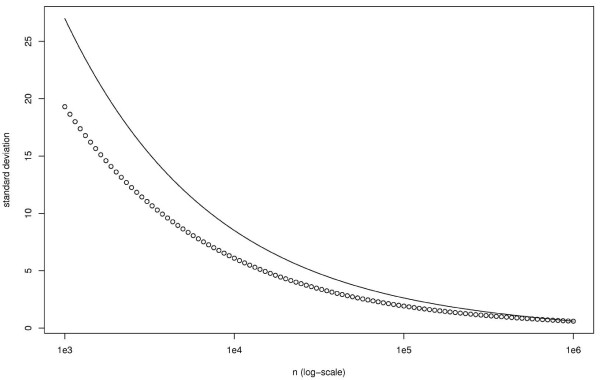
**Comparison of *σ*(*n*) and **σ˜
 MathType@MTEF@5@5@+=feaafiart1ev1aaatCvAUfKttLearuWrP9MDH5MBPbIqV92AaeXatLxBI9gBaebbnrfifHhDYfgasaacH8akY=wiFfYdH8Gipec8Eeeu0xXdbba9frFj0=OqFfea0dXdd9vqai=hGuQ8kuc9pgc9s8qqaq=dirpe0xb9q8qiLsFr0=vr0=vr0dc8meaabaqaciaacaGaaeqabaqabeGadaaakeaaiiGacuWFdpWCgaacaaaa@2E85@**(*n*)**. The circles reprensent *σ*(*n*) and the solid line σ˜
 MathType@MTEF@5@5@+=feaafiart1ev1aaatCvAUfKttLearuWrP9MDH5MBPbIqV92AaeXatLxBI9gBaebbnrfifHhDYfgasaacH8akY=wiFfYdH8Gipec8Eeeu0xXdbba9frFj0=OqFfea0dXdd9vqai=hGuQ8kuc9pgc9s8qqaq=dirpe0xb9q8qiLsFr0=vr0=vr0dc8meaabaqaciaacaGaaeqabaqabeGadaaakeaaiiGacuWFdpWCgaacaaaa@2E85@(*n*). *n*_∞ _= 10^6 ^have been used to compute the value of *A *and *B*. *N*_obs _= 1221 and ℓ = 10 000.

We can see on fig. 3 that σ˜
 MathType@MTEF@5@5@+=feaafiart1ev1aaatCvAUfKttLearuWrP9MDH5MBPbIqV92AaeXatLxBI9gBaebbnrfifHhDYfgasaacH8akY=wiFfYdH8Gipec8Eeeu0xXdbba9frFj0=OqFfea0dXdd9vqai=hGuQ8kuc9pgc9s8qqaq=dirpe0xb9q8qiLsFr0=vr0=vr0dc8meaabaqaciaacaGaaeqabaqabeGadaaakeaaiiGacuWFdpWCgaacaaaa@2E85@ is not a very good approximation of *σ *for small *n*, but, as the approximation is far easier to compute (and trivial to invert) than the true value, this can be useful when we need to compute a minimum length *n *to obtain a given *σ*.

We also see on the same figure that *σ *grows rapidly when *n *decreases. For example, we get *σ *≃ 20 for *n *= 5000 (while equation (35) gives *S *≃ 264.4).

As we consider here a binary alphabet (*k *= 2) and a first order Markov model (*m *= 1) we have only *k*^*m*^(*k *- 1) = 2 parameters to estimate with a sample of size *n *= 5000 (so we have 2500 sample per parameter). Although this situation seems quite comfortable, the sensitivity to parameter estimation appears in fact to be so large that we could have a factor 10^40 ^between the true p-value and its estimate.

#### Practical case

We have seen with our first example that our approximation works very well in a simple case. Will this hold with more practical cases?

To answer this question, let us consider the following experimental design:

• one pattern: *W *= acgtacgt;

• two genomes: *Escherichia coli *K12 (ℓ = *n *= 4639675) and *Mycoplasma genitalium *(ℓ = *n *= 580076);

• five Markov orders: *m *= 1 to *m *= 5 (larger *m *are not considered since the computation of **C **becomes then intractable).

As the sequence lengths and compositions of the two considered genomes differ a lot, we have to take a different value of *N*_obs _for each organism: *N*_obs _= 30 for *M. genitalium *and *N*_obs _= 150 for *E. coli*. Proceeding as indicated in section "simulations", we use the algorithm 1 for each experiment.

**Table 1:** Comparison of theoretical and empirical pattern statistic mean and standard deviation on *Escherichia coli *K12.

*m*	*S*	*σ*	S^ MathType@MTEF@5@5@+=feaafiart1ev1aaatCvAUfKttLearuWrP9MDH5MBPbIqV92AaeXatLxBI9gBaebbnrfifHhDYfgasaacH8akY=wiFfYdH8Gipec8Eeeu0xXdbba9frFj0=OqFfea0dXdd9vqai=hGuQ8kuc9pgc9s8qqaq=dirpe0xb9q8qiLsFr0=vr0=vr0dc8meaabaqaciaacaGaaeqabaqabeGadaaakeaacuWGtbWugaqcaaaa@2DEB@	σ^ MathType@MTEF@5@5@+=feaafiart1ev1aaatCvAUfKttLearuWrP9MDH5MBPbIqV92AaeXatLxBI9gBaebbnrfifHhDYfgasaacH8akY=wiFfYdH8Gipec8Eeeu0xXdbba9frFj0=OqFfea0dXdd9vqai=hGuQ8kuc9pgc9s8qqaq=dirpe0xb9q8qiLsFr0=vr0=vr0dc8meaabaqaciaacaGaaeqabaqabeGadaaakeaaiiGacuWFdpWCgaqcaaaa@2E86@
1	35.57	0.28	35.57	0.27
2	31.61	0.49	31.60	0.50
3	46.75	1.04	46.77	1.03
4	45.33	1.74	45.32	1.81
5	62.27	3.45	62.36	3.34

We consider the pattern *W *= acgtacgt with *N*_obs _= 150. The sequence length is ℓ = 4639675, we use an order *m *Markov model and a sample of size *M *= 1 000.

**Table 2:** Comparison of theoretical and empirical pattern statistic mean and standard deviation on *Mycoplasma genitalium*.

*m*	*S*	*σ*	S^ MathType@MTEF@5@5@+=feaafiart1ev1aaatCvAUfKttLearuWrP9MDH5MBPbIqV92AaeXatLxBI9gBaebbnrfifHhDYfgasaacH8akY=wiFfYdH8Gipec8Eeeu0xXdbba9frFj0=OqFfea0dXdd9vqai=hGuQ8kuc9pgc9s8qqaq=dirpe0xb9q8qiLsFr0=vr0=vr0dc8meaabaqaciaacaGaaeqabaqabeGadaaakeaacuWGtbWugaqcaaaa@2DEB@	σ^ MathType@MTEF@5@5@+=feaafiart1ev1aaatCvAUfKttLearuWrP9MDH5MBPbIqV92AaeXatLxBI9gBaebbnrfifHhDYfgasaacH8akY=wiFfYdH8Gipec8Eeeu0xXdbba9frFj0=OqFfea0dXdd9vqai=hGuQ8kuc9pgc9s8qqaq=dirpe0xb9q8qiLsFr0=vr0=vr0dc8meaabaqaciaacaGaaeqabaqabeGadaaakeaaiiGacuWFdpWCgaqcaaaa@2E86@
1	42.48	0.38	42.47	0.40
2	44.62	0.78	44.62	0.81
3	55.96	1.49	56.02	1.52
4	55.06	3.39	55.48	3.48
5	56.49	10.35	57.21*	9.09*

We consider the pattern *W *= acgtacgt with *N*_obs _= 30. The sequence length is ℓ = 580 076, we use an order *m *Markov model and a sample of size *M *= 1 000. (*) for 123 terms in the sample we got *P *(**N**) = 0 and hence, *S*_**N **_was not computed.

**Algorithm 1 **simulations for one experiment in the practical case

1: estimate the order *m *parameter *π *(and *μ*) from the original sequence. Although these parameters are estimated, they are considered as the true parameters;

2: compute *S *= -log_10 _ℙ(*N *≥ *N*_obs_);

3: compute *σ *using approximation (23)

4: **for ***j *= 1 ... 1 000 **do**

5: draw a random sequence *Y *= *Y*_1 _... *Y*_*n *_according to and order *m *stationary Markov model of parameter *π*;

6: compute **N **the frequency vector of all size *m *and size *m *+ 1 words in *Y*;

7: compute *S*^*j *^= *S*_**N **_= -log_10 _ℙ(*N *≥ *N*_obs_);

8: **end for**

9: compute S^
 MathType@MTEF@5@5@+=feaafiart1ev1aaatCvAUfKttLearuWrP9MDH5MBPbIqV92AaeXatLxBI9gBaebbnrfifHhDYfgasaacH8akY=wiFfYdH8Gipec8Eeeu0xXdbba9frFj0=OqFfea0dXdd9vqai=hGuQ8kuc9pgc9s8qqaq=dirpe0xb9q8qiLsFr0=vr0=vr0dc8meaabaqaciaacaGaaeqabaqabeGadaaakeaacuWGtbWugaqcaaaa@2DEB@ (resp. σ^
 MathType@MTEF@5@5@+=feaafiart1ev1aaatCvAUfKttLearuWrP9MDH5MBPbIqV92AaeXatLxBI9gBaebbnrfifHhDYfgasaacH8akY=wiFfYdH8Gipec8Eeeu0xXdbba9frFj0=OqFfea0dXdd9vqai=hGuQ8kuc9pgc9s8qqaq=dirpe0xb9q8qiLsFr0=vr0=vr0dc8meaabaqaciaacaGaaeqabaqabeGadaaakeaaiiGacuWFdpWCgaqcaaaa@2E86@) the mean (resp. standard deviation) of the sample *S*^1^,..., *S*^*j*^.

We can see on table 1 the results for *E. coli*. For each Markov model considered, our approximation of *σ *is very close to the empiric ones and, as with figure 1, the Gaussian distribution fit well to the empiric one (data not shown). Table 2 shows the same behaviour with *M. genitalium *except for *m *= 5 where σ^
 MathType@MTEF@5@5@+=feaafiart1ev1aaatCvAUfKttLearuWrP9MDH5MBPbIqV92AaeXatLxBI9gBaebbnrfifHhDYfgasaacH8akY=wiFfYdH8Gipec8Eeeu0xXdbba9frFj0=OqFfea0dXdd9vqai=hGuQ8kuc9pgc9s8qqaq=dirpe0xb9q8qiLsFr0=vr0=vr0dc8meaabaqaciaacaGaaeqabaqabeGadaaakeaaiiGacuWFdpWCgaqcaaaa@2E86@ differs slightly more than in the other cases from its theoretical value. To understand this phenomenon, let us first recall the expression of *P*(**N**) for *m *= 5 using equation (15):

P(N)=N1(agctac)×N1(gctacg)×N1(ctacgt)(ℓ−m+1)×N0(gctac)×N0(ctacg)
 MathType@MTEF@5@5@+=feaafiart1ev1aaatCvAUfKttLearuWrP9MDH5MBPbIqV92AaeXatLxBI9gBaebbnrfifHhDYfgasaacH8akY=wiFfYdH8Gipec8Eeeu0xXdbba9frFj0=OqFfea0dXdd9vqai=hGuQ8kuc9pgc9s8qqaq=dirpe0xb9q8qiLsFr0=vr0=vr0dc8meaabaqaciaacaGaaeqabaqabeGadaaakeaacqWGqbaucqGGOaakieqacqWFobGtcqGGPaqkcqGH9aqpdaWcaaqaaiab=5eaonaaBaaaleaacqaIXaqmaeqaaOGaeiikaGIaeeyyaeMaee4zaCMaee4yamMaeeiDaqNaeeyyaeMaee4yamMaeiykaKIaey41aqRae8Nta40aaSbaaSqaaiabigdaXaqabaGccqGGOaakcqqGNbWzcqqGJbWycqqG0baDcqqGHbqycqqGJbWycqqGNbWzcqGGPaqkcqGHxdaTcqWFobGtdaWgaaWcbaGaeGymaedabeaakiabcIcaOiabbogaJjabbsha0jabbggaHjabbogaJjabbEgaNjabbsha0jabcMcaPaqaaiabcIcaOiabloriSjabgkHiTiabd2gaTjabgUcaRiabigdaXiabcMcaPiabgEna0kab=5eaonaaBaaaleaacqaIWaamaeqaaOGaeiikaGIaee4zaCMaee4yamMaeeiDaqNaeeyyaeMaee4yamMaeiykaKIaey41aqRae8Nta40aaSbaaSqaaiabicdaWaqabaGccqGGOaakcqqGJbWycqqG0baDcqqGHbqycqqGJbWycqqGNbWzcqGGPaqkaaaaaa@7A52@

and as ℙ(**N**_1 _(agctac) = 0) ≃ 2.26 × 10^-6^, ℙ(**N**_1 _(gctacg) = 0) ≃ 1.35 × 10^-1 ^and ℙ(**N**_1 _(ctacgt) = 0) ≃ 1.24 × 10^-4 ^we will have *P*(**N**) = 0 roughly 14% of the time. This happened 123 times in our sample of size 1 000, each time preventing to compute *S*_**N**_. The sample is hence biased and S^
 MathType@MTEF@5@5@+=feaafiart1ev1aaatCvAUfKttLearuWrP9MDH5MBPbIqV92AaeXatLxBI9gBaebbnrfifHhDYfgasaacH8akY=wiFfYdH8Gipec8Eeeu0xXdbba9frFj0=OqFfea0dXdd9vqai=hGuQ8kuc9pgc9s8qqaq=dirpe0xb9q8qiLsFr0=vr0=vr0dc8meaabaqaciaacaGaaeqabaqabeGadaaakeaacuWGtbWugaqcaaaa@2DEB@ and σ^
 MathType@MTEF@5@5@+=feaafiart1ev1aaatCvAUfKttLearuWrP9MDH5MBPbIqV92AaeXatLxBI9gBaebbnrfifHhDYfgasaacH8akY=wiFfYdH8Gipec8Eeeu0xXdbba9frFj0=OqFfea0dXdd9vqai=hGuQ8kuc9pgc9s8qqaq=dirpe0xb9q8qiLsFr0=vr0=vr0dc8meaabaqaciaacaGaaeqabaqabeGadaaakeaaiiGacuWFdpWCgaqcaaaa@2E86@ are therefore not accurate.

What happen now if we use another statistical method to compute the pattern statistics. As the binomial approximation is supposed to be close to the exact solution, we expect the standard deviation obtained with other statistical methods to remain close to *σ*. In table 3, we compare the empirical results using binomial approximations (like above) but also compound Poisson or large deviations approximations. Both empirical means and standard deviations are close to the theoretical ones thus validating the method.

**Table 3:** Comparison of theoretical and empirical pattern statistics mean and deviation on *Mycoplasma genitalium*.

theoretical		binomial		compound Poisson		large deviations	
*S*	*σ*	S^ MathType@MTEF@5@5@+=feaafiart1ev1aaatCvAUfKttLearuWrP9MDH5MBPbIqV92AaeXatLxBI9gBaebbnrfifHhDYfgasaacH8akY=wiFfYdH8Gipec8Eeeu0xXdbba9frFj0=OqFfea0dXdd9vqai=hGuQ8kuc9pgc9s8qqaq=dirpe0xb9q8qiLsFr0=vr0=vr0dc8meaabaqaciaacaGaaeqabaqabeGadaaakeaacuWGtbWugaqcaaaa@2DEB@	σ^ MathType@MTEF@5@5@+=feaafiart1ev1aaatCvAUfKttLearuWrP9MDH5MBPbIqV92AaeXatLxBI9gBaebbnrfifHhDYfgasaacH8akY=wiFfYdH8Gipec8Eeeu0xXdbba9frFj0=OqFfea0dXdd9vqai=hGuQ8kuc9pgc9s8qqaq=dirpe0xb9q8qiLsFr0=vr0=vr0dc8meaabaqaciaacaGaaeqabaqabeGadaaakeaaiiGacuWFdpWCgaqcaaaa@2E86@	S^ MathType@MTEF@5@5@+=feaafiart1ev1aaatCvAUfKttLearuWrP9MDH5MBPbIqV92AaeXatLxBI9gBaebbnrfifHhDYfgasaacH8akY=wiFfYdH8Gipec8Eeeu0xXdbba9frFj0=OqFfea0dXdd9vqai=hGuQ8kuc9pgc9s8qqaq=dirpe0xb9q8qiLsFr0=vr0=vr0dc8meaabaqaciaacaGaaeqabaqabeGadaaakeaacuWGtbWugaqcaaaa@2DEB@	σ^ MathType@MTEF@5@5@+=feaafiart1ev1aaatCvAUfKttLearuWrP9MDH5MBPbIqV92AaeXatLxBI9gBaebbnrfifHhDYfgasaacH8akY=wiFfYdH8Gipec8Eeeu0xXdbba9frFj0=OqFfea0dXdd9vqai=hGuQ8kuc9pgc9s8qqaq=dirpe0xb9q8qiLsFr0=vr0=vr0dc8meaabaqaciaacaGaaeqabaqabeGadaaakeaaiiGacuWFdpWCgaqcaaaa@2E86@	S^ MathType@MTEF@5@5@+=feaafiart1ev1aaatCvAUfKttLearuWrP9MDH5MBPbIqV92AaeXatLxBI9gBaebbnrfifHhDYfgasaacH8akY=wiFfYdH8Gipec8Eeeu0xXdbba9frFj0=OqFfea0dXdd9vqai=hGuQ8kuc9pgc9s8qqaq=dirpe0xb9q8qiLsFr0=vr0=vr0dc8meaabaqaciaacaGaaeqabaqabeGadaaakeaacuWGtbWugaqcaaaa@2DEB@	σ^ MathType@MTEF@5@5@+=feaafiart1ev1aaatCvAUfKttLearuWrP9MDH5MBPbIqV92AaeXatLxBI9gBaebbnrfifHhDYfgasaacH8akY=wiFfYdH8Gipec8Eeeu0xXdbba9frFj0=OqFfea0dXdd9vqai=hGuQ8kuc9pgc9s8qqaq=dirpe0xb9q8qiLsFr0=vr0=vr0dc8meaabaqaciaacaGaaeqabaqabeGadaaakeaaiiGacuWFdpWCgaqcaaaa@2E86@
55.96	1.49	56.05	1.47	55.42	1.45	54.27	1.43

We consider the pattern *W *= acgtacgt with *N*_obs _= 30. The sequence length is ℓ = 580076, we use an order *m *= 3 Markov model and a sample of size *M *= 1 000. The pattern statistics are computed (from left to right) through binomial, compound Poisson or large deviations approximations.

### Choice of a Markov model order

Through the computation of *σ *we can measure the sensitivity of pattern statistics to parameter estimations. A very natural question is then, how this variability could affect a pattern statistic study, and, as this variability grows with the Markov model order, how to choose this parameter.

We propose here to consider the case of a very simple pattern study: we want to find the 100 most over-represented octamers (DNA words of size 8) in a given genome. Assuming the true parameter *π *(and hence *μ*) is known, we can compute REF = {*W*_1_,..., *W*_100_}, the list of these words (ordered by decreasing statistics, so that the most over-represented one is the first one).

For each estimates μ^
 MathType@MTEF@5@5@+=feaafiart1ev1aaatCvAUfKttLearuWrP9MDH5MBPbIqV92AaeXatLxBI9gBaebbnrfifHhDYfgasaacH8akY=wiFfYdH8Gipec8Eeeu0xXdbba9frFj0=OqFfea0dXdd9vqai=hGuQ8kuc9pgc9s8qqaq=dirpe0xb9q8qiLsFr0=vr0=vr0dc8meaabaqaciaacaGaaeqabaqabeGadaaakeaaiiGacuWF8oqBgaqcaaaa@2E79@ and π^
 MathType@MTEF@5@5@+=feaafiart1ev1aaatCvAUfKttLearuWrP9MDH5MBPbIqV92AaeXatLxBI9gBaebbnrfifHhDYfgasaacH8akY=wiFfYdH8Gipec8Eeeu0xXdbba9frFj0=OqFfea0dXdd9vqai=hGuQ8kuc9pgc9s8qqaq=dirpe0xb9q8qiLsFr0=vr0=vr0dc8meaabaqaciaacaGaaeqabaqabeGadaaakeaaiiGacuWFapaCgaqcaaaa@2E80@, we can compute REF_
 MathType@MTEF@5@5@+=feaafiart1ev1aaatCvAUfKttLearuWrP9MDH5MBPbIqV92AaeXatLxBI9gBaebbnrfifHhDYfgasaacH8akY=wiFfYdH8Gipec8Eeeu0xXdbba9frFj0=OqFfea0dXdd9vqai=hGuQ8kuc9pgc9s8qqaq=dirpe0xb9q8qiLsFr0=vr0=vr0dc8meaabaqaciaacaGaaeqabaqabeGadaaakeaadaqiaaqaaiabbkfasjabbweafjabbAeagbGaayPadaaaaa@30BD@ the 100 most over-represented octamers in the genome using the statistic S^
 MathType@MTEF@5@5@+=feaafiart1ev1aaatCvAUfKttLearuWrP9MDH5MBPbIqV92AaeXatLxBI9gBaebbnrfifHhDYfgasaacH8akY=wiFfYdH8Gipec8Eeeu0xXdbba9frFj0=OqFfea0dXdd9vqai=hGuQ8kuc9pgc9s8qqaq=dirpe0xb9q8qiLsFr0=vr0=vr0dc8meaabaqaciaacaGaaeqabaqabeGadaaakeaacuWGtbWugaqcaaaa@2DEB@ and compare it to the truth. In order to do so, we first compute the true positive rate (TP rate) defined by the rate of common words in REF_
 MathType@MTEF@5@5@+=feaafiart1ev1aaatCvAUfKttLearuWrP9MDH5MBPbIqV92AaeXatLxBI9gBaebbnrfifHhDYfgasaacH8akY=wiFfYdH8Gipec8Eeeu0xXdbba9frFj0=OqFfea0dXdd9vqai=hGuQ8kuc9pgc9s8qqaq=dirpe0xb9q8qiLsFr0=vr0=vr0dc8meaabaqaciaacaGaaeqabaqabeGadaaakeaadaqiaaqaaiabbkfasjabbweafjabbAeagbGaayPadaaaaa@30BD@ and REF, and the rank accordance rate (RA rate) defined by the Kendall's tau [[15], Chapter 13] between *S *and S^
 MathType@MTEF@5@5@+=feaafiart1ev1aaatCvAUfKttLearuWrP9MDH5MBPbIqV92AaeXatLxBI9gBaebbnrfifHhDYfgasaacH8akY=wiFfYdH8Gipec8Eeeu0xXdbba9frFj0=OqFfea0dXdd9vqai=hGuQ8kuc9pgc9s8qqaq=dirpe0xb9q8qiLsFr0=vr0=vr0dc8meaabaqaciaacaGaaeqabaqabeGadaaakeaacuWGtbWugaqcaaaa@2DEB@ ranks of {REF_
 MathType@MTEF@5@5@+=feaafiart1ev1aaatCvAUfKttLearuWrP9MDH5MBPbIqV92AaeXatLxBI9gBaebbnrfifHhDYfgasaacH8akY=wiFfYdH8Gipec8Eeeu0xXdbba9frFj0=OqFfea0dXdd9vqai=hGuQ8kuc9pgc9s8qqaq=dirpe0xb9q8qiLsFr0=vr0=vr0dc8meaabaqaciaacaGaaeqabaqabeGadaaakeaadaqiaaqaaiabbkfasjabbweafjabbAeagbGaayPadaaaaa@30BD@ ∪ REF}. Such statistic is in the range [-1,1] and has the value 1 for the complete rank accordance and the value -1 for the complete rank discordance.

As in the section "practical case", we consider two genomes: *Escherichia coli *K12 (ℓ = *n *= 4639675) and *Mycoplasma genitalium *(ℓ = *n *= 580076). For each Markov model order *m *from 1 to 6, we estimate *π *on the sequence (by maximum of likelihood), compute the REF list and then draw a sample of REF_
 MathType@MTEF@5@5@+=feaafiart1ev1aaatCvAUfKttLearuWrP9MDH5MBPbIqV92AaeXatLxBI9gBaebbnrfifHhDYfgasaacH8akY=wiFfYdH8Gipec8Eeeu0xXdbba9frFj0=OqFfea0dXdd9vqai=hGuQ8kuc9pgc9s8qqaq=dirpe0xb9q8qiLsFr0=vr0=vr0dc8meaabaqaciaacaGaaeqabaqabeGadaaakeaadaqiaaqaaiabbkfasjabbweafjabbAeagbGaayPadaaaaa@30BD@ from which we get estimates for the expectation of TP and RA rates.

Results are given in tables 4 and 5. We can see that, surprisingly, the TP rate could be very low even for long genome such as *E. coli *when high order Markov model (*m *= 6) are used. Of course, these rates are even worse on *M. genitalium *whose genome is ten times smaller than the first one. It is also clear that the RA rate is more affected by the variability induced by parameter estimation than the TP rate.

**Table 4:** Mean true positive rate and rank accordance rate in *Escherichia coli *K12.

Markov order	1	2	3	4	5	6
TP rate	99.0%	98.0%	97.9%	94.4%	82.1%	47.6%
RA rate	99.0%	95.5%	91.5%	83.9%	68.0%	36.5%
× 10^3^	383.33	95.83	23.96	5.99	1.50	0.37

Both quantities are estimated with 1 000 simulations. We consider the 1 00 most over-represented octamers, the sequence length is ℓ = 4639675. The last row gives the sample size per free parameter (length *n *of the sequence divided by the number *k*^*m*^(*k *- 1) of parameters).

**Table 5:** Mean true positive rate and rank accordance rate in *Mycoplasma genitalium*.

Markov order	1	2	3	4	5	6
TP rate	95.5%	93.6%	90.4%	81.8%	66.0%	25.0%
RA rate	92.6%	85.4%	79.8%	66.5%	45.1%	11.0%
× 10^3^	48.33	12.08	3.02	0.76	0.19	0.05

Both quantities are estimated with 1 000 simulations. We consider the 1 00 most over-represented octamers, the sequence length is ℓ = 580076. The last row gives the sample size per free parameter (length *n *of the sequence divided by the number *k*^*m*^(*k *- 1) of parameters).

Based on these results, we conclude that our pattern study requires a sample size per free parameter of at least a few thousands if we want reliable results. In our examples this has for consequence that the Markov order should not be greater than 4 (or 5 at the very most) for *E. coli *and 3 (or 4 at the very most) on *M. genitalium *without resulting in important errors.

## Conclusion

The delta-method allows us to approximate the distribution of S^
 MathType@MTEF@5@5@+=feaafiart1ev1aaatCvAUfKttLearuWrP9MDH5MBPbIqV92AaeXatLxBI9gBaebbnrfifHhDYfgasaacH8akY=wiFfYdH8Gipec8Eeeu0xXdbba9frFj0=OqFfea0dXdd9vqai=hGuQ8kuc9pgc9s8qqaq=dirpe0xb9q8qiLsFr0=vr0=vr0dc8meaabaqaciaacaGaaeqabaqabeGadaaakeaacuWGtbWugaqcaaaa@2DEB@ by a Gaussian distribution. This first requires to compute the expectation and covariance matrix of frequencies and then to study the derivative of a function which is specific of the method used to compute the pattern statistics. In the case of the binomial approximations, we have found an explicit expression of *σ *the standard deviation of S^
 MathType@MTEF@5@5@+=feaafiart1ev1aaatCvAUfKttLearuWrP9MDH5MBPbIqV92AaeXatLxBI9gBaebbnrfifHhDYfgasaacH8akY=wiFfYdH8Gipec8Eeeu0xXdbba9frFj0=OqFfea0dXdd9vqai=hGuQ8kuc9pgc9s8qqaq=dirpe0xb9q8qiLsFr0=vr0=vr0dc8meaabaqaciaacaGaaeqabaqabeGadaaakeaacuWGtbWugaqcaaaa@2DEB@.

It is clear that our approximation of *σ *using the delta-method relies one two major assumptions: 1) the distribution of **N **is Gaussian; 2) *F*^+ ^is regular enough (*e.g*. not too steep) around **E**. When *m *grows, **E **closes to the boundary of the definition range of *F*^+ ^hence degrading assumption 2. Moreover, it is well known that Gaussian approximations for word frequencies become weaker when the expected numbers of their occurrences become smaller, thus degrading assumption 1. It is therefore obvious that our approximation of *σ *will get less and less reliable as *m *grows.

However, the approximation of *σ *has been validated through simulations and appears to be very reliable (even for *m *= 5 or 6). As pattern statistics computed through binomial approximations are close to the exact statistics [8], the value of *σ *should not differ a lot when another statistical method is used. We have compared our approximations to the empiric distribution obtained using compound Poisson and large deviations approximations and, as expected, our approximations remains quite reliable even for these statistical methods.

The variability due to parameter estimation is of course related to the Markov model order *m *and to the size *k *of the alphabet (as we have *k*^*m*+1 ^parameters for this model) and to the length *n *of the sequence used for this estimation. For example, considering an order *m *= 6 model with *n *= 4639675 *(Escherichia coli *K12 complete genome) requires to estimate 3 × 4^6 ^= 4096 free parameters which results roughly in 400 observation per free parameter. Although this situation seems quite comfortable, we have seen with our simulations that it leads an unacceptable variability for pattern statistics.

As literature often advices to use the highest possible Markov order for a given pattern problem (which means *m *= *h *- 2 for pattern of size *h*) it is easy to understand that such a practice could have very detrimental effects on the computed statistics unless huge data are available for estimation purpose. Even if we consider the more reasonable attitude to choose *m *using the classical framework of model selection (*e.g*. using the Akaike Information Criterion – AIC –) we get *m *= 5 for *Mycoplasma genitalium *and *m *= 6 for *Escherichia coli *K12 hence resulting in both cases in the same catastrophic results in terms of false positive and even worse ones in terms of ranking.

Moreover, we assumed here that our model was homogeneous all along the considered sequences. This is obviously completely false when complete genomes are considered. So it is more likely that the sample size *n *would be far smaller than a million on classical pattern studies (even of human genomes for example). As a result, the variability we pointed out in this paper will have a considerable detrimental effect on most studies unless the Markov order is carefully set.

In order to do so, we advice to compute our approximation of *σ *each time a pattern statistic is produced and then to evaluate, either by simulation (like in this paper) or by a theoretical work the impact of this variability on the considered study.

## Competing interests

The author declares that he has no competing interests.

## Appendix A

We give here the expression of the covariance matrix **C **introduced in section "distribution of **N **= (**N**_0_, **N**_1_)". The sequence *Y *(of length *n*) is generated by an homogeneous, stationary and ergodic order *m *Markov model of parameter *π *and stationary distribution *μ*. We want to compute the covariance of the vector **N **of random frequencies of size *m *and *m *+ 1 words.

For any word *w *(of size *h*_*w*_), we introduce the following notation for *h*_*w *_≤ *i *≤ *n*

Ii(w)=I{w endinpositioni}=I{Yi−hw+1i=w}     (45)
 MathType@MTEF@5@5@+=feaafiart1ev1aaatCvAUfKttLearuWrP9MDH5MBPbIqV92AaeXatLxBI9gBamrtHrhAL1wy0L2yHvtyaeHbnfgDOvwBHrxAJfwnaebbnrfifHhDYfgasaacH8akY=wiFfYdH8Gipec8Eeeu0xXdbba9frFj0=OqFfea0dXdd9vqai=hGuQ8kuc9pgc9s8qqaq=dirpe0xb9q8qiLsFr0=vr0=vr0dc8meaabaqaciaacaGaaeqabaWaaeGaeaaakeaacqWGjbqsdaWgaaWcbaGaemyAaKgabeaakiabcIcaOiabdEha3jabcMcaPiabg2da9mrr1ngBPrwtHrhAYaqehuuDJXwAKbstHrhAGq1DVbacfaGae8hIWN0aaSbaaSqaaiabcUha7jabdEha3jabbccaGGqaaiab+vgaLjab+5gaUjab+rgaKjab+bcaGiab+LgaPjab+5gaUjab+bcaGiab+bhaWjab+9gaVjab+nhaZjab+LgaPjab+rha0jab+LgaPjab+9gaVjab+5gaUjab+bcaGiabdMgaPjabc2ha9bqabaGccqGH9aqpcqWFicFsdaWgaaWcbaGaei4EaSNaemywaK1aa0baaWqaaiabdMgaPjabgkHiTiabdIgaOnaaBaaabaGaem4DaChabeaacqGHRaWkcqaIXaqmaeaacqWGPbqAaaWccqGH9aqpcqWG3bWDcqGG9bqFaeqaaOGaaCzcaiaaxMaacqGGOaakcqaI0aancqaI1aqncqGGPaqkaaa@7BBF@

where Yij
 MathType@MTEF@5@5@+=feaafiart1ev1aaatCvAUfKttLearuWrP9MDH5MBPbIqV92AaeXatLxBI9gBaebbnrfifHhDYfgasaacH8akY=wiFfYdH8Gipec8Eeeu0xXdbba9frFj0=OqFfea0dXdd9vqai=hGuQ8kuc9pgc9s8qqaq=dirpe0xb9q8qiLsFr0=vr0=vr0dc8meaabaqaciaacaGaaeqabaqabeGadaaakeaacqWGzbqwdaqhaaWcbaGaemyAaKgabaGaemOAaOgaaaaa@30CC@ = *Y*_*i *_... *Y*_*j *_for all *i *≤ *j*. If *h*_*w *_≥ *m*, we denote by

p(w)=μ(w1m)Π(w1m,wm+1)...Π(whw−mh−1,wh)     (46)
 MathType@MTEF@5@5@+=feaafiart1ev1aaatCvAUfKttLearuWrP9MDH5MBPbIqV92AaeXatLxBI9gBaebbnrfifHhDYfgasaacH8akY=wiFfYdH8Gipec8Eeeu0xXdbba9frFj0=OqFfea0dXdd9vqai=hGuQ8kuc9pgc9s8qqaq=dirpe0xb9q8qiLsFr0=vr0=vr0dc8meaabaqaciaacaGaaeqabaqabeGadaaakeaacqWGWbaCcqGGOaakcqWG3bWDcqGGPaqkcqGH9aqpiiGacqWF8oqBcqGGOaakcqWG3bWDdaqhaaWcbaGaeGymaedabaGaemyBa0gaaOGaeiykaKIaeuiOdaLaeiikaGIaem4DaC3aa0baaSqaaiabigdaXaqaaiabd2gaTbaakiabcYcaSiabdEha3naaBaaaleaacqWGTbqBcqGHRaWkcqaIXaqmaeqaaOGaeiykaKIaeiOla4IaeiOla4IaeiOla4IaeuiOdaLaeiikaGIaem4DaC3aa0baaSqaaiabdIgaOnaaBaaameaacqWG3bWDaeqaaSGaeyOeI0IaemyBa0gabaGaemiAaGMaeyOeI0IaeGymaedaaOGaeiilaWIaem4DaC3aaSbaaSqaaiabdIgaObqabaGccqGGPaqkcaWLjaGaaCzcaiabcIcaOiabisda0iabiAda2iabcMcaPaaa@5F8B@

the probability to see one occurrence of *w *at a given position in the sequence. At last, if we consider another word *v *(of size *h*_*v *_= *m*) and if *h*_*w *_= *m*, we denote by

Πδ(v,w)=∑x∈Aδp(vxw)     (47)
 MathType@MTEF@5@5@+=feaafiart1ev1aaatCvAUfKttLearuWrP9MDH5MBPbIqV92AaeXatLxBI9gBamrtHrhAL1wy0L2yHvtyaeHbnfgDOvwBHrxAJfwnaebbnrfifHhDYfgasaacH8akY=wiFfYdH8Gipec8Eeeu0xXdbba9frFj0=OqFfea0dXdd9vqai=hGuQ8kuc9pgc9s8qqaq=dirpe0xb9q8qiLsFr0=vr0=vr0dc8meaabaqaciaacaGaaeqabaWaaeGaeaaakeaacqqHGoaudaWgaaWcbaacciGae8hTdqgabeaakiabcIcaOiabdAha2jabcYcaSiabdEha3jabcMcaPiabg2da9maaqafabaGaemiCaaNaeiikaGIaemODayNaemiEaGNaem4DaCNaeiykaKcaleaacqWG4baEcqGHiiIZimaacqGFaeFqdaahaaadbeqaaiab=r7aKbaaaSqab0GaeyyeIuoakiaaxMaacaWLjaGaeiikaGIaeGinaqJaeG4naCJaeiykaKcaaa@556F@

the probability to see occurrences of *v *and *w *separated by a gap of length δ.

For any words *v *an *w *(to simplify, we suppose that *h*_*v *_≥ *h*_*w*_) then, for all δ ∈ ℤ
 MathType@MTEF@5@5@+=feaafiart1ev1aaatCvAUfKttLearuWrP9MDH5MBPbIqV92AaeXatLxBI9gBaebbnrfifHhDYfgasaacH8akY=wiFfYdH8Gipec8Eeeu0xXdbba9frFj0=OqFfea0dXdd9vqai=hGuQ8kuc9pgc9s8qqaq=dirpe0xb9q8qiLsFr0=vr0=vr0dc8meaabaqaciaacaGaaeqabaqabeGadaaakeaatuuDJXwAK1uy0HMmaeHbfv3ySLgzG0uy0HgiuD3BaGabaiab=rsiAbaa@3772@ and

max(*h*_*v*_, *h*_*w *_- δ) ≤ *i *≤ min(*n*, *n *- δ) we have

E
 MathType@MTEF@5@5@+=feaafiart1ev1aaatCvAUfKttLearuWrP9MDH5MBPbIqV92AaeXatLxBI9gBaebbnrfifHhDYfgasaacH8akY=wiFfYdH8Gipec8Eeeu0xXdbba9frFj0=OqFfea0dXdd9vqai=hGuQ8kuc9pgc9s8qqaq=dirpe0xb9q8qiLsFr0=vr0=vr0dc8meaabaqaciaacaGaaeqabaqabeGadaaakeaatuuDJXwAK1uy0HMmaeHbfv3ySLgzG0uy0HgiuD3BaGabaiab=ri8fbaa@388C@ [*I*_*i *_(*v*) *I*_*i*+δ _(*w*)] = *D*_δ _(*v*, *w*)     (48)

which do not depend on *i*.

It is therefore easy to show that

E[N(v)N(w)]=∑i=hvn∑δ=hw−in−iDδ(v,w)     (49)=∑δ=hw−nn−hvNδDδ(v,w)(50)=M(v,w)+O(v,m)(51)
 MathType@MTEF@5@5@+=feaafiart1ev1aaatCvAUfKttLearuWrP9MDH5MBPbIqV92AaeXatLxBI9gBaebbnrfifHhDYfgasaacH8akY=wiFfYdH8Gipec8Eeeu0xXdbba9frFj0=OqFfea0dXdd9vqai=hGuQ8kuc9pgc9s8qqaq=dirpe0xb9q8qiLsFr0=vr0=vr0dc8meaabaqaciaacaGaaeqabaqabeGadaaakeGabaa9fuaabmqadiaaaeaatuuDJXwAK1uy0HMmaeHbfv3ySLgzG0uy0HgiuD3BaGabaiab=ri8fjabcUfaBHqabiab+5eaojabcIcaOiabdAha2jabcMcaPiab+5eaojabcIcaOiabdEha3jabcMcaPiabc2faDjabg2da9maaqahabaWaaabCaeaacqWGebardaWgaaWcbaacciGae0hTdqgabeaakiabcIcaOiabdAha2jabcYcaSiabdEha3jabcMcaPaWcbaGae0hTdqMaeyypa0JaemiAaG2aaSbaaWqaaiabdEha3bqabaWccqGHsislcqWGPbqAaeaacqWGUbGBcqGHsislcqWGPbqAa0GaeyyeIuoaaSqaaiabdMgaPjabg2da9iabdIgaOnaaBaaameaacqWG2bGDaeqaaaWcbaGaemOBa4ganiabggHiLdGccaWLjaGaaCzcaaqaamaabmaabaGaeGinaqJaeGyoaKdacaGLOaGaayzkaaaabaGaeyypa0ZaaabCaeaacqWGobGtdaWgaaWcbaGae0hTdqgabeaakiabdseaenaaBaaaleaacqqF0oazaeqaaOGaeiikaGIaemODayNaeiilaWIaem4DaCNaeiykaKcaleaacqqF0oazcqGH9aqpcqWGObaAdaWgaaadbaGaem4DaChabeaaliabgkHiTiabd6gaUbqaaiabd6gaUjabgkHiTiabdIgaOnaaBaaameaacqWG2bGDaeqaaaqdcqGHris5aaGcbaWaaeWaaeaacqaI1aqncqaIWaamaiaawIcacaGLPaaaaeaacqGH9aqpcqGFnbqtcqGGOaakcqWG2bGDcqGGSaalcqWG3bWDcqGGPaqkcqGHRaWkcqGFpbWtcqGGOaakcqWG2bGDcqGGSaalcqWGTbqBcqGGPaqkaeaadaqadaqaaiabiwda1iabigdaXaGaayjkaiaawMcaaaaaaaa@9BD3@

where the main part (2*n *- *h*_*v *_- *h*_*w *_+ 2 terms) is given by

M(v,w)=∑δ=hvn−hwN−δD−δ(v,w)+∑δ=hwn−hvNδDδ(v,w)     (52)
 MathType@MTEF@5@5@+=feaafiart1ev1aaatCvAUfKttLearuWrP9MDH5MBPbIqV92AaeXatLxBI9gBaebbnrfifHhDYfgasaacH8akY=wiFfYdH8Gipec8Eeeu0xXdbba9frFj0=OqFfea0dXdd9vqai=hGuQ8kuc9pgc9s8qqaq=dirpe0xb9q8qiLsFr0=vr0=vr0dc8meaabaqaciaacaGaaeqabaqabeGadaaakeaaieqacqWFnbqtcqGGOaakcqWG2bGDcqGGSaalcqWG3bWDcqGGPaqkcqGH9aqpdaaeWbqaaiabd6eaonaaBaaaleaacqGHsisliiGacqGF0oazaeqaaOGaemiraq0aaSbaaSqaaiabgkHiTiab+r7aKbqabaaabaGae4hTdqMaeyypa0JaemiAaG2aaSbaaWqaaiabdAha2bqabaaaleaacqWGUbGBcqGHsislcqWGObaAdaWgaaadbaGaem4DaChabeaaa0GaeyyeIuoakiabcIcaOiabdAha2jabcYcaSiabdEha3jabcMcaPiabgUcaRmaaqahabaGaemOta40aaSbaaSqaaiab+r7aKbqabaGccqWGebardaWgaaWcbaGae4hTdqgabeaaaeaacqGF0oazcqGH9aqpcqWGObaAdaWgaaadbaGaem4DaChabeaaaSqaaiabd6gaUjabgkHiTiabdIgaOnaaBaaameaacqWG2bGDaeqaaaqdcqGHris5aOGaeiikaGIaemODayNaeiilaWIaem4DaCNaeiykaKIaaCzcaiaaxMaacqGGOaakcqaI1aqncqaIYaGmcqGGPaqkaaa@6D1F@

and the overlapping part (*h*_*v *_+ *h*_*w *_- 1 terms) by

O(v,w)=∑δ=−hv+1hw−1NδDδ(v,w)     (53)
 MathType@MTEF@5@5@+=feaafiart1ev1aaatCvAUfKttLearuWrP9MDH5MBPbIqV92AaeXatLxBI9gBaebbnrfifHhDYfgasaacH8akY=wiFfYdH8Gipec8Eeeu0xXdbba9frFj0=OqFfea0dXdd9vqai=hGuQ8kuc9pgc9s8qqaq=dirpe0xb9q8qiLsFr0=vr0=vr0dc8meaabaqaciaacaGaaeqabaqabeGadaaakeaaieqacqWFpbWtcqGGOaakcqWG2bGDcqGGSaalcqWG3bWDcqGGPaqkcqGH9aqpdaaeWbqaaiabd6eaonaaBaaaleaaiiGacqGF0oazaeqaaOGaemiraq0aaSbaaSqaaiab+r7aKbqabaGccqGGOaakcqWG2bGDcqGGSaalcqWG3bWDcqGGPaqkaSqaaiab+r7aKjabg2da9iabgkHiTiabdIgaOnaaBaaameaacqWG2bGDaeqaaSGaey4kaSIaeGymaedabaGaemiAaG2aaSbaaWqaaiabdEha3bqabaWccqGHsislcqaIXaqma0GaeyyeIuoakiaaxMaacaWLjaGaeiikaGIaeGynauJaeG4mamJaeiykaKcaaa@5444@

and with

Nδ={n−hw+1+δδ∈[hw−n,hw−hv[n−hv+1δ∈[hw−hv,0]n−hv+1−δδ∈]0,n−hv]     (54)
 MathType@MTEF@5@5@+=feaafiart1ev1aaatCvAUfKttLearuWrP9MDH5MBPbIqV92AaeXatLxBI9gBaebbnrfifHhDYfgasaacH8akY=wiFfYdH8Gipec8Eeeu0xXdbba9frFj0=OqFfea0dXdd9vqai=hGuQ8kuc9pgc9s8qqaq=dirpe0xb9q8qiLsFr0=vr0=vr0dc8meaabaqaciaacaGaaeqabaqabeGadaaakeaacqWGobGtdaWgaaWcbaacciGae8hTdqgabeaakiabg2da9maaceqabaqbaeaabmGaaaqaaiabd6gaUjabgkHiTiabdIgaOnaaBaaaleaacqWG3bWDaeqaaOGaey4kaSIaeGymaeJaey4kaSIae8hTdqgabaGae8hTdqMaeyicI4Saei4waSLaemiAaG2aaSbaaSqaaiabdEha3bqabaGccqGHsislcqWGUbGBcqGGSaalcqWGObaAdaWgaaWcbaGaem4DaChabeaakiabgkHiTiabdIgaOnaaBaaaleaacqWG2bGDaeqaaOGaei4waSfabaGaemOBa4MaeyOeI0IaemiAaG2aaSbaaSqaaiabdAha2bqabaGccqGHRaWkcqaIXaqmaeaacqWF0oazcqGHiiIZcqGGBbWwcqWGObaAdaWgaaWcbaGaem4DaChabeaakiabgkHiTiabdIgaOnaaBaaaleaacqWG2bGDaeqaaOGaeiilaWIaeGimaaJaeiyxa0fabaGaemOBa4MaeyOeI0IaemiAaG2aaSbaaSqaaiabdAha2bqabaGccqGHRaWkcqaIXaqmcqGHsislcqWF0oazaeaacqWF0oazcqGHiiIZcqGGDbqxcqaIWaamcqGGSaalcqWGUbGBcqGHsislcqWGObaAdaWgaaWcbaGaemODayhabeaakiabc2faDbaaaiaawUhaaiaaxMaacaWLjaGaeiikaGIaeGynauJaeGinaqJaeiykaKcaaa@7F57@

As we have

**C**(*v*, *w*) = **M **(*v*, *w*) + **O **(*v*, *w*) - **E **(*v*) **E **(*w*)     (55)

the problem is hence to compute **M **and **O **for all pairs of size *m *or *m *+ 1 words. In order to simplify, we will just treat here the case of a pair of size *m *words (other cases can be derived from this special case).

For the main part we obtain

M(v,w)=∑δ=mn−mN−δμ(w)Πδ−m+1(w,v)+∑δ=mn−mNδμ(v)Πδ−m+1(v,w)     (56)
 MathType@MTEF@5@5@+=feaafiart1ev1aaatCvAUfKttLearuWrP9MDH5MBPbIqV92AaeXatLxBI9gBaebbnrfifHhDYfgasaacH8akY=wiFfYdH8Gipec8Eeeu0xXdbba9frFj0=OqFfea0dXdd9vqai=hGuQ8kuc9pgc9s8qqaq=dirpe0xb9q8qiLsFr0=vr0=vr0dc8meaabaqaciaacaGaaeqabaqabeGadaaakeaafaqadeGabaaabaacbeGae8xta0KaeiikaGIaemODayNaeiilaWIaem4DaCNaeiykaKIaeyypa0ZaaabCaeaacqWGobGtdaWgaaWcbaGaeyOeI0ccciGae4hTdqgabeaakiab+X7aTjabcIcaOiabdEha3jabcMcaPiabfc6aqnaaBaaaleaacqGF0oazcqGHsislcqWGTbqBcqGHRaWkcqaIXaqmaeqaaOGaeiikaGIaem4DaCNaeiilaWIaemODayNaeiykaKcaleaacqGF0oazcqGH9aqpcqWGTbqBaeaacqWGUbGBcqGHsislcqWGTbqBa0GaeyyeIuoaaOqaaiabgUcaRmaaqahabaGaemOta40aaSbaaSqaaiab+r7aKbqabaGccqGF8oqBcqGGOaakcqWG2bGDcqGGPaqkcqqHGoaudaWgaaWcbaGae4hTdqMaeyOeI0IaemyBa0Maey4kaSIaeGymaedabeaakiabcIcaOiabdAha2jabcYcaSiabdEha3jabcMcaPaWcbaGae4hTdqMaeyypa0JaemyBa0gabaGaemOBa4MaeyOeI0IaemyBa0ganiabggHiLdaaaOGaaCzcaiaaxMaacqGGOaakcqaI1aqncqaI2aGncqGGPaqkaaa@78C7@

(2*n *- 2*m *+ 2 terms). As *P*_*k *_(*v*, *w*) quickly converges toward *μ*(*w*) when *k *grows (convergence speed is given by *λ*^*k *^where *λ *is the magnitude of the second eigenvalue of the transition matrix Π). So there exists a rank *r *≥ *m *such as

M(v,w)≃μ(v)μ(w)∑δ=rn−m(N−δ+Nδ)+∑δ=mr−1N−δμ(w)Πδ−m+1(w,v)+∑δ=mr−1Nδμ(v)Πδ−m+1(v,w)     (57)
 MathType@MTEF@5@5@+=feaafiart1ev1aaatCvAUfKttLearuWrP9MDH5MBPbIqV92AaeXatLxBI9gBaebbnrfifHhDYfgasaacH8akY=wiFfYdH8Gipec8Eeeu0xXdbba9frFj0=OqFfea0dXdd9vqai=hGuQ8kuc9pgc9s8qqaq=dirpe0xb9q8qiLsFr0=vr0=vr0dc8meaabaqaciaacaGaaeqabaqabeGadaaakqGabeqaaW8abaacbeGae8xta0KaeiikaGIaemODayNaeiilaWIaem4DaCNaeiykaKIaeS4qISdcciGae4hVd0MaeiikaGIaemODayNaeiykaKIae4hVd0MaeiikaGIaem4DaCNaeiykaKYaaabCaeaacqGGOaakcqWGobGtdaWgaaWcbaGaeyOeI0Iae4hTdqgabeaakiabgUcaRiabd6eaonaaBaaaleaacqGF0oazaeqaaOGaeiykaKcaleaacqGF0oazcqGH9aqpcqWGYbGCaeaacqWGUbGBcqGHsislcqWGTbqBa0GaeyyeIuoaaOqaaiaaxMaacqGHRaWkdaaeWbqaaiabd6eaonaaBaaaleaacqGHsislcqGF0oazaeqaaOGae4hVd0MaeiikaGIaem4DaCNaeiykaKIaeuiOda1aaSbaaSqaaiab+r7aKjabgkHiTiabd2gaTjabgUcaRiabigdaXaqabaaabaGae4hTdqMaeyypa0JaemyBa0gabaGaemOCaiNaeyOeI0IaeGymaedaniabggHiLdGccqGGOaakcqWG3bWDcqGGSaalcqWG2bGDcqGGPaqkaeaacaWLjaGaey4kaSYaaabCaeaacqWGobGtdaWgaaWcbaGae4hTdqgabeaakiab+X7aTjabcIcaOiabdAha2jabcMcaPiabfc6aqnaaBaaaleaacqGF0oazcqGHsislcqWGTbqBcqGHRaWkcqaIXaqmaeqaaaqaaiab+r7aKjabg2da9iabd2gaTbqaaiabdkhaYjabgkHiTiabigdaXaqdcqGHris5aOGaeiikaGIaemODayNaeiilaWIaem4DaCNaeiykaKIaaCzcaiaaxMaacqGGOaakcqaI1aqncqaI3aWncqGGPaqkaaaa@981D@

which has only 2*r *- 2*m *+ 1 terms.

And for the overlapping part we get

O(v,w)=N0×μ(v)×I{v=w}+∑δ=1m−1N−δ×p(wvm−δ+1m)×I{v1m−δ=w1+δm}+∑δ=1m−1Nδ×p(vwm−δ+1m)×I{v1+δm=w1m−δ}     (58)
 MathType@MTEF@5@5@+=feaafiart1ev1aaatCvAUfKttLearuWrP9MDH5MBPbIqV92AaeXatLxBI9gBaebbnrfifHhDYfgasaacH8akY=wiFfYdH8Gipec8Eeeu0xXdbba9frFj0=OqFfea0dXdd9vqai=hGuQ8kuc9pgc9s8qqaq=dirpe0xb9q8qiLsFr0=vr0=vr0dc8meaabaqaciaacaGaaeqabaqabeGadaaakqGabeqaaaNabaacbeGae83ta8KaeiikaGIaemODayNaeiilaWIaem4DaCNaeiykaKIaeyypa0JaemOta40aaSbaaSqaaiabicdaWaqabaGccqGHxdaTiiGacqGF8oqBcqGGOaakcqWG2bGDcqGGPaqkcqGHxdaTtuuDJXwAK1uy0HMmaeHbfv3ySLgzG0uy0HgiuD3BaGabaiab9Hi8jnaaBaaaleaacqGG7bWEcqWG2bGDcqGH9aqpcqWG3bWDcqGG9bqFaeqaaaGcbaGaaCzcaiabgUcaRmaaqahabaGaemOta40aaSbaaSqaaiabgkHiTiab+r7aKbqabaaabaGae4hTdqMaeyypa0JaeGymaedabaGaemyBa0MaeyOeI0IaeGymaedaniabggHiLdGccqGHxdaTcqWGWbaCcqGGOaakcqWG3bWDcqWG2bGDdaqhaaWcbaGaemyBa0MaeyOeI0Iae4hTdqMaey4kaSIaeGymaedabaGaemyBa0gaaOGaeiykaKIaey41aqRae0hIWN0aaSbaaSqaamaacmqabaGaemODay3aa0baaWqaaiabigdaXaqaaiabd2gaTjabgkHiTiabes7aKbaaliabg2da9iabdEha3naaDaaameaacqaIXaqmcqGHRaWkcqGF0oazaeaacqWGTbqBaaaaliaawUhacaGL9baaaeqaaaGcbaGaaCzcaiabgUcaRmaaqahabaGaemOta40aaSbaaSqaaiab+r7aKbqabaGccqGHxdaTcqWGWbaCcqGGOaakcqWG2bGDcqWG3bWDdaqhaaWcbaGaemyBa0MaeyOeI0Iae4hTdqMaey4kaSIaeGymaedabaGaemyBa0gaaOGaeiykaKcaleaacqGF0oazcqGH9aqpcqaIXaqmaeaacqWGTbqBcqGHsislcqaIXaqma0GaeyyeIuoakiabgEna0kab9Hi8jnaaBaaaleaadaGadeqaaiabdAha2naaDaaameaacqaIXaqmcqGHRaWkcqGF0oazaeaacqWGTbqBaaWccqGH9aqpcqWG3bWDdaqhaaadbaGaeGymaedabaGaemyBa0MaeyOeI0Iae4hTdqgaaaWccaGL7bGaayzFaaaabeaakiaaxMaacaWLjaGaeiikaGIaeGynauJaeGioaGJaeiykaKcaaaa@BCF0@

which has 2*m *+ 1 terms.

So the overall complexity for the computation of one term of **C **is hence *O*(*r*) where the value of *r *is directly connected to the magnitude *λ *of the second eigenvalue of the transition matrix.

In the particular case of an order one Markov model (*m *= 1), we give here the complete expressions of **M **and **O**.

For all *a*, *b*, *c*, *d *∈ A
 MathType@MTEF@5@5@+=feaafiart1ev1aaatCvAUfKttLearuWrP9MDH5MBPbIqV92AaeXatLxBI9gBamrtHrhAL1wy0L2yHvtyaeHbnfgDOvwBHrxAJfwnaebbnrfifHhDYfgasaacH8akY=wiFfYdH8Gipec8Eeeu0xXdbba9frFj0=OqFfea0dXdd9vqai=hGuQ8kuc9pgc9s8qqaq=dirpe0xb9q8qiLsFr0=vr0=vr0dc8meaabaqaciaacaGaaeqabaWaaeGaeaaakeaaimaacqWFaeFqaaa@3821@, we have

M(a,b)≃(n−r+1)(n−r)μ(a)μ(b)+∑δ=1r−1(n−δ)(μ(b)Πδ(b,a)+μ(a)Πδ(a,b))     (59)
 MathType@MTEF@5@5@+=feaafiart1ev1aaatCvAUfKttLearuWrP9MDH5MBPbIqV92AaeXatLxBI9gBaebbnrfifHhDYfgasaacH8akY=wiFfYdH8Gipec8Eeeu0xXdbba9frFj0=OqFfea0dXdd9vqai=hGuQ8kuc9pgc9s8qqaq=dirpe0xb9q8qiLsFr0=vr0=vr0dc8meaabaqaciaacaGaaeqabaqabeGadaaakqaabeqaaGqabiab=1eanjabcIcaOiabdggaHjabcYcaSiabdkgaIjabcMcaPiabloKi7iabcIcaOiabd6gaUjabgkHiTiabdkhaYjabgUcaRiabigdaXiabcMcaPiabcIcaOiabd6gaUjabgkHiTiabdkhaYjabcMcaPGGaciab+X7aTjabcIcaOiabdggaHjabcMcaPiab+X7aTjabcIcaOiabdkgaIjabcMcaPaqaaiabgUcaRmaaqahabaGaeiikaGIaemOBa4MaeyOeI0Iae4hTdqMaeiykaKYaaeWaaeaacqGF8oqBcqGGOaakcqWGIbGycqGGPaqkcqqHGoaudaahaaWcbeqaaiab+r7aKbaakiabcIcaOiabdkgaIjabcYcaSiabdggaHjabcMcaPiabgUcaRiab+X7aTjabcIcaOiabdggaHjabcMcaPiabfc6aqnaaCaaaleqabaGae4hTdqgaaOGaeiikaGIaemyyaeMaeiilaWIaemOyaiMaeiykaKcacaGLOaGaayzkaaGaaCzcaiaaxMaacqGGOaakcqaI1aqncqaI5aqocqGGPaqkaSqaaiab+r7aKjabg2da9iabigdaXaqaaiabdkhaYjabgkHiTiabigdaXaqdcqGHris5aaaaaa@7BAA@

**O **(*a*, *b*) = *nμ*(*a*) I
 MathType@MTEF@5@5@+=feaafiart1ev1aaatCvAUfKttLearuWrP9MDH5MBPbIqV92AaeXatLxBI9gBaebbnrfifHhDYfgasaacH8akY=wiFfYdH8Gipec8Eeeu0xXdbba9frFj0=OqFfea0dXdd9vqai=hGuQ8kuc9pgc9s8qqaq=dirpe0xb9q8qiLsFr0=vr0=vr0dc8meaabaqaciaacaGaaeqabaqabeGadaaakeaatuuDJXwAK1uy0HMmaeHbfv3ySLgzG0uy0HgiuD3BaGabaiab=Hi8jbaa@3894@_{*a *= *b*} _    (60)

M(ab,c)Π(a,b)≃(n−r)(n−r−1)μ(a)μ(c)+∑δ=1r−1(n−δ−1)(μ(c)Πδ(c,a)+μ(a)Πδ(b,c))     (61)
 MathType@MTEF@5@5@+=feaafiart1ev1aaatCvAUfKttLearuWrP9MDH5MBPbIqV92AaeXatLxBI9gBaebbnrfifHhDYfgasaacH8akY=wiFfYdH8Gipec8Eeeu0xXdbba9frFj0=OqFfea0dXdd9vqai=hGuQ8kuc9pgc9s8qqaq=dirpe0xb9q8qiLsFr0=vr0=vr0dc8meaabaqaciaacaGaaeqabaqabeGadaaakqaabeqaamaalaaabaacbeGae8xta0KaeiikaGIaemyyaeMaemOyaiMaeiilaWIaem4yamMaeiykaKcabaGaeuiOdaLaeiikaGIaemyyaeMaeiilaWIaemOyaiMaeiykaKcaaiabloKi7iabcIcaOiabd6gaUjabgkHiTiabdkhaYjabcMcaPiabcIcaOiabd6gaUjabgkHiTiabdkhaYjabgkHiTiabigdaXiabcMcaPGGaciab+X7aTjabcIcaOiabdggaHjabcMcaPiab+X7aTjabcIcaOiabdogaJjabcMcaPaqaaiabgUcaRmaaqahabaGaeiikaGIaemOBa4MaeyOeI0Iae4hTdqMaeyOeI0IaeGymaeJaeiykaKYaaeWaaeaacqGF8oqBcqGGOaakcqWGJbWycqGGPaqkcqqHGoaudaahaaWcbeqaaiab+r7aKbaakiabcIcaOiabdogaJjabcYcaSiabdggaHjabcMcaPiabgUcaRiab+X7aTjabcIcaOiabdggaHjabcMcaPiabfc6aqnaaCaaaleqabaGae4hTdqgaaOGaeiikaGIaemOyaiMaeiilaWIaem4yamMaeiykaKcacaGLOaGaayzkaaGaaCzcaiaaxMaacqGGOaakcqaI2aGncqaIXaqmcqGGPaqkaSqaaiab+r7aKjabg2da9iabigdaXaqaaiabdkhaYjabgkHiTiabigdaXaqdcqGHris5aaaaaa@8595@

O(ab,c)Π(a,b)=(n−1)μ(a)(I{a=c}+I{b=c})     (62)
 MathType@MTEF@5@5@+=feaafiart1ev1aaatCvAUfKttLearuWrP9MDH5MBPbIqV92AaeXatLxBI9gBaebbnrfifHhDYfgasaacH8akY=wiFfYdH8Gipec8Eeeu0xXdbba9frFj0=OqFfea0dXdd9vqai=hGuQ8kuc9pgc9s8qqaq=dirpe0xb9q8qiLsFr0=vr0=vr0dc8meaabaqaciaacaGaaeqabaqabeGadaaakeaadaWcaaqaaGqabiab=9eapjabcIcaOiabdggaHjabdkgaIjabcYcaSiabdogaJjabcMcaPaqaaiabfc6aqjabcIcaOiabdggaHjabcYcaSiabdkgaIjabcMcaPaaacqGH9aqpcqGGOaakcqWGUbGBcqGHsislcqaIXaqmcqGGPaqkiiGacqGF8oqBcqGGOaakcqWGHbqycqGGPaqkcqGGOaaktuuDJXwAK1uy0HMmaeHbfv3ySLgzG0uy0HgiuD3BaGabaiab9Hi8jnaaBaaaleaacqGG7bWEcqWGHbqycqGH9aqpcqWGJbWycqGG9bqFaeqaaOGaey4kaSIae0hIWN0aaSbaaSqaaiabcUha7jabdkgaIjabg2da9iabdogaJjabc2ha9bqabaGccqGGPaqkcaWLjaGaaCzcaiabcIcaOiabiAda2iabikdaYiabcMcaPaaa@690B@

M(ab,cd)Π(a,b)Π(c,d)≃(n−r−1)(n−r−2)μ(a)μ(c)+∑δ=1r−1(n−δ−2)(μ(c)Πδ(d,a)+μ(a)Πδ(b,c))     (63)
 MathType@MTEF@5@5@+=feaafiart1ev1aaatCvAUfKttLearuWrP9MDH5MBPbIqV92AaeXatLxBI9gBaebbnrfifHhDYfgasaacH8akY=wiFfYdH8Gipec8Eeeu0xXdbba9frFj0=OqFfea0dXdd9vqai=hGuQ8kuc9pgc9s8qqaq=dirpe0xb9q8qiLsFr0=vr0=vr0dc8meaabaqaciaacaGaaeqabaqabeGadaaakqaabeqaamaalaaabaacbeGae8xta0KaeiikaGIaemyyaeMaemOyaiMaeiilaWIaem4yamMaemizaqMaeiykaKcabaGaeuiOdaLaeiikaGIaemyyaeMaeiilaWIaemOyaiMaeiykaKIaeuiOdaLaeiikaGIaem4yamMaeiilaWIaemizaqMaeiykaKcaaiabloKi7iabcIcaOiabd6gaUjabgkHiTiabdkhaYjabgkHiTiabigdaXiabcMcaPiabcIcaOiabd6gaUjabgkHiTiabdkhaYjabgkHiTiabikdaYiabcMcaPGGaciab+X7aTjabcIcaOiabdggaHjabcMcaPiab+X7aTjabcIcaOiabdogaJjabcMcaPaqaaiabgUcaRmaaqahabaGaeiikaGIaemOBa4MaeyOeI0Iae4hTdqMaeyOeI0IaeGOmaiJaeiykaKYaaeWaaeaacqGF8oqBcqGGOaakcqWGJbWycqGGPaqkcqqHGoaudaahaaWcbeqaaiab+r7aKbaakiabcIcaOiabdsgaKjabcYcaSiabdggaHjabcMcaPiabgUcaRiab+X7aTjabcIcaOiabdggaHjabcMcaPiabfc6aqnaaCaaaleqabaGae4hTdqgaaOGaeiikaGIaemOyaiMaeiilaWIaem4yamMaeiykaKcacaGLOaGaayzkaaGaaCzcaiaaxMaacqGGOaakcqaI2aGncqaIZaWmcqGGPaqkaSqaaiab+r7aKjabg2da9iabigdaXaqaaiabdkhaYjabgkHiTiabigdaXaqdcqGHris5aaaaaa@8F7D@

O(ab,cd)Π(a,b)=(n−1)μ(a)I{ab=cd}+(n−2)Π(c,d)(μ(c)I{a=d}+μ(a)I{b=c})     (64)
 MathType@MTEF@5@5@+=feaafiart1ev1aaatCvAUfKttLearuWrP9MDH5MBPbIqV92AaeXatLxBI9gBaebbnrfifHhDYfgasaacH8akY=wiFfYdH8Gipec8Eeeu0xXdbba9frFj0=OqFfea0dXdd9vqai=hGuQ8kuc9pgc9s8qqaq=dirpe0xb9q8qiLsFr0=vr0=vr0dc8meaabaqaciaacaGaaeqabaqabeGadaaakqaabeqaamaalaaabaacbeGae83ta8KaeiikaGIaemyyaeMaemOyaiMaeiilaWIaem4yamMaemizaqMaeiykaKcabaGaeuiOdaLaeiikaGIaemyyaeMaeiilaWIaemOyaiMaeiykaKcaaiabg2da9iabcIcaOiabd6gaUjabgkHiTiabigdaXiabcMcaPGGaciab+X7aTjabcIcaOiabdggaHjabcMcaPmrr1ngBPrwtHrhAYaqeguuDJXwAKbstHrhAGq1DVbaceaGae0hIWN0aaSbaaSqaaiabcUha7jabdggaHjabdkgaIjabg2da9iabdogaJjabdsgaKjabc2ha9bqabaaakeaacqGHRaWkcqGGOaakcqWGUbGBcqGHsislcqaIYaGmcqGGPaqkcqqHGoaucqGGOaakcqWGJbWycqGGSaalcqWGKbazcqGGPaqkdaqadaqaaiab+X7aTjabcIcaOiabdogaJjabcMcaPiab9Hi8jnaaBaaaleaacqGG7bWEcqWGHbqycqGH9aqpcqWGKbazcqGG9bqFaeqaaOGaey4kaSIae4hVd0MaeiikaGIaemyyaeMaeiykaKIae0hIWN0aaSbaaSqaaiabcUha7jabdkgaIjabg2da9iabdogaJjabc2ha9bqabaaakiaawIcacaGLPaaacaWLjaGaaCzcaiabcIcaOiabiAda2iabisda0iabcMcaPaaaaa@8BDE@

With the example given in section "validation" we get for the expectation

E0t
 MathType@MTEF@5@5@+=feaafiart1ev1aaatCvAUfKttLearuWrP9MDH5MBPbIqV92AaeXatLxBI9gBaebbnrfifHhDYfgasaacH8akY=wiFfYdH8Gipec8Eeeu0xXdbba9frFj0=OqFfea0dXdd9vqai=hGuQ8kuc9pgc9s8qqaq=dirpe0xb9q8qiLsFr0=vr0=vr0dc8meaabaqaciaacaGaaeqabaqabeGadaaakeaaieqacqWFfbqrdaqhaaWcbaGaeGimaadabaGaemiDaqhaaaaa@3051@ = [4615.4 5384.6]     (65)

and

E1t
 MathType@MTEF@5@5@+=feaafiart1ev1aaatCvAUfKttLearuWrP9MDH5MBPbIqV92AaeXatLxBI9gBaebbnrfifHhDYfgasaacH8akY=wiFfYdH8Gipec8Eeeu0xXdbba9frFj0=OqFfea0dXdd9vqai=hGuQ8kuc9pgc9s8qqaq=dirpe0xb9q8qiLsFr0=vr0=vr0dc8meaabaqaciaacaGaaeqabaqabeGadaaakeaaieqacqWFfbqrdaqhaaWcbaGaeGymaedabaGaemiDaqhaaaaa@3053@ = [1384.5 3230.4 3230.4 2153.6]     (66)

The magnitude of the second eigenvalue of Π is *λ *= 0.3, then rank *r *= 19 give a relative error < 10 ^-10 ^and we get for the covariance

C0,0=[1338.28−1338.28−1338.281338.28]     (67)
 MathType@MTEF@5@5@+=feaafiart1ev1aaatCvAUfKttLearuWrP9MDH5MBPbIqV92AaeXatLxBI9gBaebbnrfifHhDYfgasaacH8akY=wiFfYdH8Gipec8Eeeu0xXdbba9frFj0=OqFfea0dXdd9vqai=hGuQ8kuc9pgc9s8qqaq=dirpe0xb9q8qiLsFr0=vr0=vr0dc8meaabaqaciaacaGaaeqabaqabeGadaaakeaaieqacqWFdbWqdaWgaaWcbaGaeGimaaJaeiilaWIaeGimaadabeaakiabg2da9maadmaabaqbaeqabiGaaaqaaiabigdaXiabiodaZiabiodaZiabiIda4iabc6caUiabikdaYiabiIda4aqaaiabgkHiTiabigdaXiabiodaZiabiodaZiabiIda4iabc6caUiabikdaYiabiIda4aqaaiabgkHiTiabigdaXiabiodaZiabiodaZiabiIda4iabc6caUiabikdaYiabiIda4aqaaiabigdaXiabiodaZiabiodaZiabiIda4iabc6caUiabikdaYiabiIda4aaaaiaawUfacaGLDbaacaWLjaGaaCzcaiabcIcaOiabiAda2iabiEda3iabcMcaPaaa@5529@

C1,0=[1146.9191.2191.2−1529.2−1146.9−191.2−191.21529.2]     (68)
 MathType@MTEF@5@5@+=feaafiart1ev1aaatCvAUfKttLearuWrP9MDH5MBPbIqV92AaeXatLxBI9gBaebbnrfifHhDYfgasaacH8akY=wiFfYdH8Gipec8Eeeu0xXdbba9frFj0=OqFfea0dXdd9vqai=hGuQ8kuc9pgc9s8qqaq=dirpe0xb9q8qiLsFr0=vr0=vr0dc8meaabaqaciaacaGaaeqabaqabeGadaaakeaaieqacqWFdbWqdaWgaaWcbaGaeGymaeJaeiilaWIaeGimaadabeaakiabg2da9maadmaabaqbaeqabiabaaaabaGaeGymaeJaeGymaeJaeGinaqJaeGOnayJaeiOla4IaeGyoaKdabaGaeGymaeJaeGyoaKJaeGymaeJaeiOla4IaeGOmaidabaGaeGymaeJaeGyoaKJaeGymaeJaeiOla4IaeGOmaidabaGaeyOeI0IaeGymaeJaeGynauJaeGOmaiJaeGyoaKJaeiOla4IaeGOmaidabaGaeyOeI0IaeGymaeJaeGymaeJaeGinaqJaeGOnayJaeiOla4IaeGyoaKdabaGaeyOeI0IaeGymaeJaeGyoaKJaeGymaeJaeiOla4IaeGOmaidabaGaeyOeI0IaeGymaeJaeGyoaKJaeGymaeJaeiOla4IaeGOmaidabaGaeGymaeJaeGynauJaeGOmaiJaeGyoaKJaeiOla4IaeGOmaidaaaGaay5waiaaw2faaiaaxMaacaWLjaGaeiikaGIaeGOnayJaeGioaGJaeiykaKcaaa@6605@

and

C1,1=[1536.8−390.0−390.0−756.9−390.0581.2581.0−772.2−390.0581.0581.2−772.2−756.9−772.2−772.22301.4]     (69)
 MathType@MTEF@5@5@+=feaafiart1ev1aaatCvAUfKttLearuWrP9MDH5MBPbIqV92AaeXatLxBI9gBaebbnrfifHhDYfgasaacH8akY=wiFfYdH8Gipec8Eeeu0xXdbba9frFj0=OqFfea0dXdd9vqai=hGuQ8kuc9pgc9s8qqaq=dirpe0xb9q8qiLsFr0=vr0=vr0dc8meaabaqaciaacaGaaeqabaqabeGadaaakeaaieqacqWFdbWqdaWgaaWcbaGaeGymaeJaeiilaWIaeGymaedabeaakiabg2da9maadmaabaqbaeqabqabaaaaaeaacqaIXaqmcqaI1aqncqaIZaWmcqaI2aGncqGGUaGlcqaI4aaoaeaacqGHsislcqaIZaWmcqaI5aqocqaIWaamcqGGUaGlcqaIWaamaeaacqGHsislcqaIZaWmcqaI5aqocqaIWaamcqGGUaGlcqaIWaamaeaacqGHsislcqaI3aWncqaI1aqncqaI2aGncqGGUaGlcqaI5aqoaeaacqGHsislcqaIZaWmcqaI5aqocqaIWaamcqGGUaGlcqaIWaamaeaacqaI1aqncqaI4aaocqaIXaqmcqGGUaGlcqaIYaGmaeaacqaI1aqncqaI4aaocqaIXaqmcqGGUaGlcqaIWaamaeaacqGHsislcqaI3aWncqaI3aWncqaIYaGmcqGGUaGlcqaIYaGmaeaacqGHsislcqaIZaWmcqaI5aqocqaIWaamcqGGUaGlcqaIWaamaeaacqaI1aqncqaI4aaocqaIXaqmcqGGUaGlcqaIWaamaeaacqaI1aqncqaI4aaocqaIXaqmcqGGUaGlcqaIYaGmaeaacqGHsislcqaI3aWncqaI3aWncqaIYaGmcqGGUaGlcqaIYaGmaeaacqGHsislcqaI3aWncqaI1aqncqaI2aGncqGGUaGlcqaI5aqoaeaacqGHsislcqaI3aWncqaI3aWncqaIYaGmcqGGUaGlcqaIYaGmaeaacqGHsislcqaI3aWncqaI3aWncqaIYaGmcqGGUaGlcqaIYaGmaeaacqaIYaGmcqaIZaWmcqaIWaamcqaIXaqmcqGGUaGlcqaI0aanaaaacaGLBbGaayzxaaGaaCzcaiaaxMaacqGGOaakcqaI2aGncqaI5aqocqGGPaqkaaa@8FB3@

## Appendix B

The beta function is defined by

β(a,b)=∫01ta−1(1−t)b−1dt     (70)
 MathType@MTEF@5@5@+=feaafiart1ev1aaatCvAUfKttLearuWrP9MDH5MBPbIqV92AaeXatLxBI9gBaebbnrfifHhDYfgasaacH8akY=wiFfYdH8Gipec8Eeeu0xXdbba9frFj0=OqFfea0dXdd9vqai=hGuQ8kuc9pgc9s8qqaq=dirpe0xb9q8qiLsFr0=vr0=vr0dc8meaabaqaciaacaGaaeqabaqabeGadaaakeaaiiGacqWFYoGycqGGOaakcqWGHbqycqGGSaalcqWGIbGycqGGPaqkcqGH9aqpdaWdXaqaaiabdsha0naaCaaaleqabaGaemyyaeMaeyOeI0IaeGymaedaaOGaeiikaGIaeGymaeJaeyOeI0IaemiDaqNaeiykaKYaaWbaaSqabeaacqWGIbGycqGHsislcqaIXaqmaaGccqWGKbazcqWG0baDcaWLjaGaaCzcaiabcIcaOiabiEda3iabicdaWiabcMcaPaWcbaGaeGimaadabaGaeGymaedaniabgUIiYdaaaa@4D5E@

for all *a*, *b *> 0. The incomplete beta function for all *x *∈ [0,1] is then defined by

β(x,a,b)=∫0xta−1(1−t)b−1dt     (71)
 MathType@MTEF@5@5@+=feaafiart1ev1aaatCvAUfKttLearuWrP9MDH5MBPbIqV92AaeXatLxBI9gBaebbnrfifHhDYfgasaacH8akY=wiFfYdH8Gipec8Eeeu0xXdbba9frFj0=OqFfea0dXdd9vqai=hGuQ8kuc9pgc9s8qqaq=dirpe0xb9q8qiLsFr0=vr0=vr0dc8meaabaqaciaacaGaaeqabaqabeGadaaakeaaiiGacqWFYoGycqGGOaakcqWG4baEcqGGSaalcqWGHbqycqGGSaalcqWGIbGycqGGPaqkcqGH9aqpdaWdXaqaaiabdsha0naaCaaaleqabaGaemyyaeMaeyOeI0IaeGymaedaaaqaaiabicdaWaqaaiabdIha4bqdcqGHRiI8aOGaeiikaGIaeGymaeJaeyOeI0IaemiDaqNaeiykaKYaaWbaaSqabeaacqWGIbGycqGHsislcqaIXaqmaaGccqWGKbazcqWG0baDcaWLjaGaaCzcaiabcIcaOiabiEda3iabigdaXiabcMcaPaaa@5037@

and

β−(x,a,b)=β(a,b)−β(x,a,b)     (72)=∫x1ta−1(1−t)b−1dt(73)
 MathType@MTEF@5@5@+=feaafiart1ev1aaatCvAUfKttLearuWrP9MDH5MBPbIqV92AaeXatLxBI9gBaebbnrfifHhDYfgasaacH8akY=wiFfYdH8Gipec8Eeeu0xXdbba9frFj0=OqFfea0dXdd9vqai=hGuQ8kuc9pgc9s8qqaq=dirpe0xb9q8qiLsFr0=vr0=vr0dc8meaabaqaciaacaGaaeqabaqabeGadaaakeGabaabfuaabmqaciaaaeaaiiGacqWFYoGydaahaaWcbeqaaiabgkHiTaaakiabcIcaOiabdIha4jabcYcaSiabdggaHjabcYcaSiabdkgaIjabcMcaPiabg2da9iab=j7aIjabcIcaOiabdggaHjabcYcaSiabdkgaIjabcMcaPiabgkHiTiab=j7aIjabcIcaOiabdIha4jabcYcaSiabdggaHjabcYcaSiabdkgaIjabcMcaPiaaxMaacaWLjaaabaWaaeWaaeaacqaI3aWncqaIYaGmaiaawIcacaGLPaaaaeaacqGH9aqpdaWdXaqaaiabdsha0naaCaaaleqabaGaemyyaeMaeyOeI0IaeGymaedaaaqaaiabdIha4bqaaiabigdaXaqdcqGHRiI8aOGaeiikaGIaeGymaeJaeyOeI0IaemiDaqNaeiykaKYaaWbaaSqabeaacqWGIbGycqGHsislcqaIXaqmaaGccqWGKbazcqWG0baDaeaadaqadaqaaiabiEda3iabiodaZaGaayjkaiaawMcaaaaaaaa@66A5@

Using a continued fraction representation, these functions can be quickly numerically evaluated in *O*(max⁡(a,b)
 MathType@MTEF@5@5@+=feaafiart1ev1aaatCvAUfKttLearuWrP9MDH5MBPbIqV92AaeXatLxBI9gBaebbnrfifHhDYfgasaacH8akY=wiFfYdH8Gipec8Eeeu0xXdbba9frFj0=OqFfea0dXdd9vqai=hGuQ8kuc9pgc9s8qqaq=dirpe0xb9q8qiLsFr0=vr0=vr0dc8meaabaqaciaacaGaaeqabaqabeGadaaakeaadaGcaaqaaiGbc2gaTjabcggaHjabcIha4jabcIcaOiabdggaHjabcYcaSiabdkgaIjabcMcaPaWcbeaaaaa@3617@) in the worst case [15, Chapter 6].

A great interest of this function is that it is connected to the cumulative distribution function of a binomial distribution by the following relation:

ℙ(ℬ(n,p)≥k)=β(p,k,n−k+1)β(k,n−k+1)     (74)
 MathType@MTEF@5@5@+=feaafiart1ev1aaatCvAUfKttLearuWrP9MDH5MBPbIqV92AaeXatLxBI9gBamrtHrhAL1wy0L2yHvtyaeHbnfgDOvwBHrxAJfwnaebbnrfifHhDYfgasaacH8akY=wiFfYdH8Gipec8Eeeu0xXdbba9frFj0=OqFfea0dXdd9vqai=hGuQ8kuc9pgc9s8qqaq=dirpe0xb9q8qiLsFr0=vr0=vr0dc8meaabaqaciaacaGaaeqabaWaaeGaeaaakeaatuuDJXwAK1uy0HMmaeXbfv3ySLgzG0uy0HgiuD3BaGqbaiab=LriqjabcIcaOGWaaiab+XsicjabcIcaOiabd6gaUjabcYcaSiabdchaWjabcMcaPiabgwMiZkabdUgaRjabcMcaPiabg2da9maalaaabaacciGae0NSdiMaeiikaGIaemiCaaNaeiilaWIaem4AaSMaeiilaWIaemOBa4MaeyOeI0Iaem4AaSMaey4kaSIaeGymaeJaeiykaKcabaGae0NSdiMaeiikaGIaem4AaSMaeiilaWIaemOBa4MaeyOeI0Iaem4AaSMaey4kaSIaeGymaeJaeiykaKcaaiaaxMaacaWLjaGaeiikaGIaeG4naCJaeGinaqJaeiykaKcaaa@6AD6@

with (*n*, *k*) ∈ ℕ* × ℕ, 0 ≤ *k *≤ *n *and *p *∈ [0,1].

Finally, let us remark that the incomplete beta function is differentiable in *x *and that

∂β(x,a,b)∂x=xa−1(1−x)b−1     (75)
 MathType@MTEF@5@5@+=feaafiart1ev1aaatCvAUfKttLearuWrP9MDH5MBPbIqV92AaeXatLxBI9gBaebbnrfifHhDYfgasaacH8akY=wiFfYdH8Gipec8Eeeu0xXdbba9frFj0=OqFfea0dXdd9vqai=hGuQ8kuc9pgc9s8qqaq=dirpe0xb9q8qiLsFr0=vr0=vr0dc8meaabaqaciaacaGaaeqabaqabeGadaaakeaadaWcaaqaaGGaciab=jGi2kab=j7aIjabcIcaOiabdIha4jabcYcaSiabdggaHjabcYcaSiabdkgaIjabcMcaPaqaaiab=jGi2kabdIha4baacqGH9aqpcqWG4baEdaahaaWcbeqaaiabdggaHjabgkHiTiabigdaXaaakiabcIcaOiabigdaXiabgkHiTiabdIha4jabcMcaPmaaCaaaleqabaGaemOyaiMaeyOeI0IaeGymaedaaOGaaCzcaiaaxMaacqGGOaakcqaI3aWncqaI1aqncqGGPaqkaaa@4D4F@

## Appendix C

We give here the complete expression of *σ *for a single pattern in the special case of an order *m *= 0 homogeneous Markov model of parameter *μ*.

The MLE of *μ *is given by

μN=N1n     (76)
 MathType@MTEF@5@5@+=feaafiart1ev1aaatCvAUfKttLearuWrP9MDH5MBPbIqV92AaeXatLxBI9gBaebbnrfifHhDYfgasaacH8akY=wiFfYdH8Gipec8Eeeu0xXdbba9frFj0=OqFfea0dXdd9vqai=hGuQ8kuc9pgc9s8qqaq=dirpe0xb9q8qiLsFr0=vr0=vr0dc8meaabaqaciaacaGaaeqabaqabeGadaaakeaaiiGacqWF8oqBdaWgaaWcbaacbeGae4Nta4eabeaakiabg2da9maalaaabaGae4Nta40aaSbaaSqaaiabigdaXaqabaaakeaacqWGUbGBaaGaaCzcaiaaxMaacqGGOaakcqaI3aWncqaI2aGncqGGPaqkaaa@3976@

where **N**_1 _is the frequency of all letters.

A Gaussian approximation gives

L
 MathType@MTEF@5@5@+=feaafiart1ev1aaatCvAUfKttLearuWrP9MDH5MBPbIqV92AaeXatLxBI9gBaebbnrfifHhDYfgasaacH8akY=wiFfYdH8Gipec8Eeeu0xXdbba9frFj0=OqFfea0dXdd9vqai=hGuQ8kuc9pgc9s8qqaq=dirpe0xb9q8qiLsFr0=vr0=vr0dc8meaabaqaciaacaGaaeqabaqabeGadaaakeaatCvAUfeBSjuyZL2yd9gzLbvyNv2CaeHbnf2C0vMCJfMCKbaceiGaa8htaaaa@394B@ (**N**_1_) ≃ N
 MathType@MTEF@5@5@+=feaafiart1ev1aaatCvAUfKttLearuWrP9MDH5MBPbIqV92AaeXatLxBI9gBamrtHrhAL1wy0L2yHvtyaeHbnfgDOvwBHrxAJfwnaebbnrfifHhDYfgasaacH8akY=wiFfYdH8Gipec8Eeeu0xXdbba9frFj0=OqFfea0dXdd9vqai=hGuQ8kuc9pgc9s8qqaq=dirpe0xb9q8qiLsFr0=vr0=vr0dc8meaabaqaciaacaGaaeqabaWaaeGaeaaakeaaimaacqWFneVtaaa@383B@ (**E**_1_, **C**_1,1_)     (77)

with **E**_1 _= *nμ *and, for all *a*, *b *∈ A
 MathType@MTEF@5@5@+=feaafiart1ev1aaatCvAUfKttLearuWrP9MDH5MBPbIqV92AaeXatLxBI9gBamrtHrhAL1wy0L2yHvtyaeHbnfgDOvwBHrxAJfwnaebbnrfifHhDYfgasaacH8akY=wiFfYdH8Gipec8Eeeu0xXdbba9frFj0=OqFfea0dXdd9vqai=hGuQ8kuc9pgc9s8qqaq=dirpe0xb9q8qiLsFr0=vr0=vr0dc8meaabaqaciaacaGaaeqabaWaaeGaeaaakeaaimaacqWFaeFqaaa@3821@,

**C**_1,1 _(*a, b*) = *nμ *(*a*) I
 MathType@MTEF@5@5@+=feaafiart1ev1aaatCvAUfKttLearuWrP9MDH5MBPbIqV92AaeXatLxBI9gBaebbnrfifHhDYfgasaacH8akY=wiFfYdH8Gipec8Eeeu0xXdbba9frFj0=OqFfea0dXdd9vqai=hGuQ8kuc9pgc9s8qqaq=dirpe0xb9q8qiLsFr0=vr0=vr0dc8meaabaqaciaacaGaaeqabaqabeGadaaakeaatuuDJXwAK1uy0HMmaeHbfv3ySLgzG0uy0HgiuD3BaGabaiab=Hi8jbaa@3894@_*a *= *b *_- *nμ*(*a*) × *nμ*(*b*)     (78)

We have also

P(N)=1nh∏a∈AN1(a)A1(a)     (79)
 MathType@MTEF@5@5@+=feaafiart1ev1aaatCvAUfKttLearuWrP9MDH5MBPbIqV92AaeXatLxBI9gBamrtHrhAL1wy0L2yHvtyaeHbnfgDOvwBHrxAJfwnaebbnrfifHhDYfgasaacH8akY=wiFfYdH8Gipec8Eeeu0xXdbba9frFj0=OqFfea0dXdd9vqai=hGuQ8kuc9pgc9s8qqaq=dirpe0xb9q8qiLsFr0=vr0=vr0dc8meaabaqaciaacaGaaeqabaWaaeGaeaaakeaacqWGqbaucqGGOaakieqacqWFobGtcqGGPaqkcqGH9aqpdaWcaaqaaiabigdaXaqaaiabd6gaUnaaCaaaleqabaGaemiAaGgaaaaakmaarafabaGae8Nta40aaSbaaSqaaiabigdaXaqabaGccqGGOaakcqWGHbqycqGGPaqkdaahaaWcbeqaaiabdgeabnaaBaaameaacqaIXaqmaeqaaSGaeiikaGIaemyyaeMaeiykaKcaaaqaaiabdggaHjabgIGioJWaaiab+bq8bbqab0Gaey4dIunakiaaxMaacaWLjaGaeiikaGIaeG4naCJaeGyoaKJaeiykaKcaaa@5594@

which implies for all *a *∈ A
 MathType@MTEF@5@5@+=feaafiart1ev1aaatCvAUfKttLearuWrP9MDH5MBPbIqV92AaeXatLxBI9gBamrtHrhAL1wy0L2yHvtyaeHbnfgDOvwBHrxAJfwnaebbnrfifHhDYfgasaacH8akY=wiFfYdH8Gipec8Eeeu0xXdbba9frFj0=OqFfea0dXdd9vqai=hGuQ8kuc9pgc9s8qqaq=dirpe0xb9q8qiLsFr0=vr0=vr0dc8meaabaqaciaacaGaaeqabaWaaeGaeaaakeaaimaacqWFaeFqaaa@3821@ that

∂P(N)∂N1(a)=A1(a)N1(a)︸G1(a)×P(N)     (80)
 MathType@MTEF@5@5@+=feaafiart1ev1aaatCvAUfKttLearuWrP9MDH5MBPbIqV92AaeXatLxBI9gBaebbnrfifHhDYfgasaacH8akY=wiFfYdH8Gipec8Eeeu0xXdbba9frFj0=OqFfea0dXdd9vqai=hGuQ8kuc9pgc9s8qqaq=dirpe0xb9q8qiLsFr0=vr0=vr0dc8meaabaqaciaacaGaaeqabaqabeGadaaakeaadaWcaaqaaiabgkGi2kabdcfaqjabcIcaOGqabiab=5eaojabcMcaPaqaaiabgkGi2kab=5eaonaaBaaaleaacqaIXaqmaeqaaOGaeiikaGIaemyyaeMaeiykaKcaaiabg2da9maalaaabaGaemyqae0aaSbaaSqaaiabigdaXaqabaGccqGGOaakcqWGHbqycqGGPaqkaeaadaagaaqaaiab=5eaonaaBaaaleaacqaIXaqmaeqaaOGaeiikaGIaemyyaeMaeiykaKcaleaacqWFhbWrdaWgaaadbaGaeGymaedabeaaliabcIcaOiabdggaHjabcMcaPaGccaGL44paaaGaey41aqRaemiuaaLaeiikaGIae8Nta4KaeiykaKIaaCzcaiaaxMaacqGGOaakcqaI4aaocqaIWaamcqGGPaqkaaa@5687@

So finally we get

σ≃Q tG1×C1,1×G1     (81)
 MathType@MTEF@5@5@+=feaafiart1ev1aaatCvAUfKttLearuWrP9MDH5MBPbIqV92AaeXatLxBI9gBaebbnrfifHhDYfgasaacH8akY=wiFfYdH8Gipec8Eeeu0xXdbba9frFj0=OqFfea0dXdd9vqai=hGuQ8kuc9pgc9s8qqaq=dirpe0xb9q8qiLsFr0=vr0=vr0dc8meaabaqaciaacaGaaeqabaqabeGadaaakeaaiiGacqWFdpWCcqWIdjYocqWGrbqudaGcaaqaaiabbccaGmaaCaaaleqabaacbiGae4hDaqhaaGqabOGae03raC0aaSbaaSqaaiabigdaXaqabaGccqGHxdaTcqqFdbWqdaWgaaWcbaGaeGymaeJaeiilaWIaeGymaedabeaakiabgEna0kab9DeahnaaBaaaleaacqaIXaqmaeqaaaqabaGccaWLjaGaaCzcaiabcIcaOiabiIda4iabigdaXiabcMcaPaaa@44E0@

where *Q *is either defined by equation (24) if the pattern is over-represented or by equation (28) if under-represented.
